# Auritus: An Open-Source Optimization Toolkit for Training and Development of Human Movement Models and Filters Using Earables

**DOI:** 10.1145/3534586

**Published:** 2022-07-07

**Authors:** SWAPNIL SAYAN SAHA, SANDEEP SINGH SANDHA, SIYOU PEI, VIVEK JAIN, ZIQI WANG, YUCHEN LI, ANKUR SARKER, MANI SRIVASTAVA

**Affiliations:** University of California - Los Angeles, USA

**Keywords:** earable, network architecture search, neural networks, machine learning, datasets, filters, human activity, head-pose, TinyML, optimization, hardware-in-the-loop

## Abstract

Smart ear-worn devices (called earables) are being equipped with various onboard sensors and algorithms, transforming earphones from simple audio transducers to multi-modal interfaces making rich inferences about human motion and vital signals. However, developing sensory applications using earables is currently quite cumbersome with several barriers in the way. First, time-series data from earable sensors incorporate information about physical phenomena in complex settings, requiring machine-learning (ML) models learned from large-scale labeled data. This is challenging in the context of earables because large-scale open-source datasets are missing. Secondly, the small size and compute constraints of earable devices make on-device integration of many existing algorithms for tasks such as human activity and head-pose estimation difficult. To address these challenges, we introduce Auritus an extendable and open-source optimization toolkit designed to enhance and replicate earable applications. Auritus serves two primary functions. *Firstly*, Auritus handles data collection, pre-processing, and labeling tasks for creating customized earable datasets using graphical tools. The system includes an open-source dataset with 2.43 million inertial samples related to head and full-body movements, consisting of 34 head poses and 9 activities from 45 volunteers. *Secondly*, Auritus provides a tightly-integrated hardware-in-the-loop (HIL) optimizer and TinyML interface to develop lightweight and real-time machine-learning (ML) models for activity detection and filters for head-pose tracking. To validate the utlity of Auritus, we showcase three sample applications, namely fall detection, spatial audio rendering, and augmented reality (AR) interfacing. Auritus recognizes activities with 91% leave 1-out test accuracy (98% test accuracy) using real-time models as small as 6-13 kB. Our models are 98-740× smaller and 3-6% more accurate over the state-of-the-art. We also estimate head pose with absolute errors as low as 5 degrees using 20kB filters, achieving up to 1.6× precision improvement over existing techniques. We make the entire system open-source so that researchers and developers can contribute to any layer of the system or rapidly prototype their applications using our dataset and algorithms.

## 1 INTRODUCTION

Earables – electronic devices that sit inside one’s ears – have undergone rapid transformation in recent years [19, 46]. From being primarily used for wireless audio capture and playback in a convenient form factor and natural placement, earables are now being transformed into devices with rich multimodal sensing capabilities, on-device local processing, and storage [19, 103]. While one could always re-purpose the existing microphone and radio transceivers already in the earables [14, 19, 26, 72], many devices have begun to add other sensors [102]. In-ear headphones and smart earbuds are now equipped with ultra-low-power inertial measurement units (IMU) [46], beamforming microphone arrays [19], temperature sensors [69], and heart-rate monitors [79]. Information from these additional sensing modalities not only enhance the primary audio functionality for purposes such as smarter spatial audio but, as recent research activity shows, can also potentially be harnessed to sense the wearer’s physical states and contexts, such as facial activity, head motion, vital signs, etc [45].

Among the current applications of earables, human activity detection [5, 38, 39, 51, 64, 75, 92] and head-pose recognition [28, 76] form the principal engineering research problem for most applications [14, 19, 54, 63, 77, 91, 96]. The natural placement of ear-worn devices can provide key information about the wearer’s movement and vital signals [19, 45]. As a result, the ability to track head motion and gait data has spawned a plethora of emerging applications. These include dead-reckoning [3, 72, 74], binaural audio rendering [102], robot control [67], authentication [19, 98], context detection [26, 43], health monitoring [32, 60, 66, 80], mixed-reality (MR), AR and tangible interfaces [26, 65, 93, 102]. These emerging applications have been shown to benefit not only general consumers but parties with special needs and interests [19]. For example, dead-reckoning and 3D spatial audio can be combined to provide localization cues for the visually-impaired [3, 14, 65, 102]. Gesture, posture, and expression monitoring have been used to regulate food intake [63], mental health [45, 54, 96], sleep [60], vital signs [32, 66, 80], and proper posture [92] in patients through mHealth. Fitness enthusiasts can use earables not only to listen to music during exercise but also monitor their performance and respiration rate [75, 77, 80]. Given the opportunities, the global earable market is projected to grow exponentially, and significant commercial ventures are already underway [19, 45, 79, 103].

### 1.1 Challenges

The bulk of emerging innovations builds upon recent advances in machine learning (ML) for sensor-based activity detection and gesture recognition [2, 11, 52, 68] because classical signal processing techniques do not yield high enough accuracy for complex activity recognition. However, the development of ML classifiers for human activity detection (as well as analytical head-pose filters) on earables face the following challenges:

- **The Absence of Large-Scale Earable Datasets and Software Tools**: The data-hungry nature of ML training demands access to large-scale earable datasets. Although abundant datasets are publicly available for smartphones and non-ear-worn wearables [4, 15, 33, 44, 61, 83, 95, 104], the relative newness of earables means that we are missing open-source and well-curated earable datasets [19]. In addition, the software tools enabling automation in the earable data collection pipeline have not received significant attention. Hence, in the context of earables, the reproducibility and rigorous benchmarking of the performance of models and algorithms remain challenging.
- **The Compute Constraints of Earables**: Earables have tight memory, power, and compute constraints [19]. For example, an earable has only 56 KB RAM and 16 MB of flash [46], compared to 4 GB of RAM and 64 GB of storage available on a smartphone [57]. As a result, directly transferring existing ML classifiers and filters for activity detection and head-pose estimation from other domains (e.g., smartphones and smartwatches) is not feasible for onboard computation on earable.

### 1.2 Contributions

**Fig. 1.**
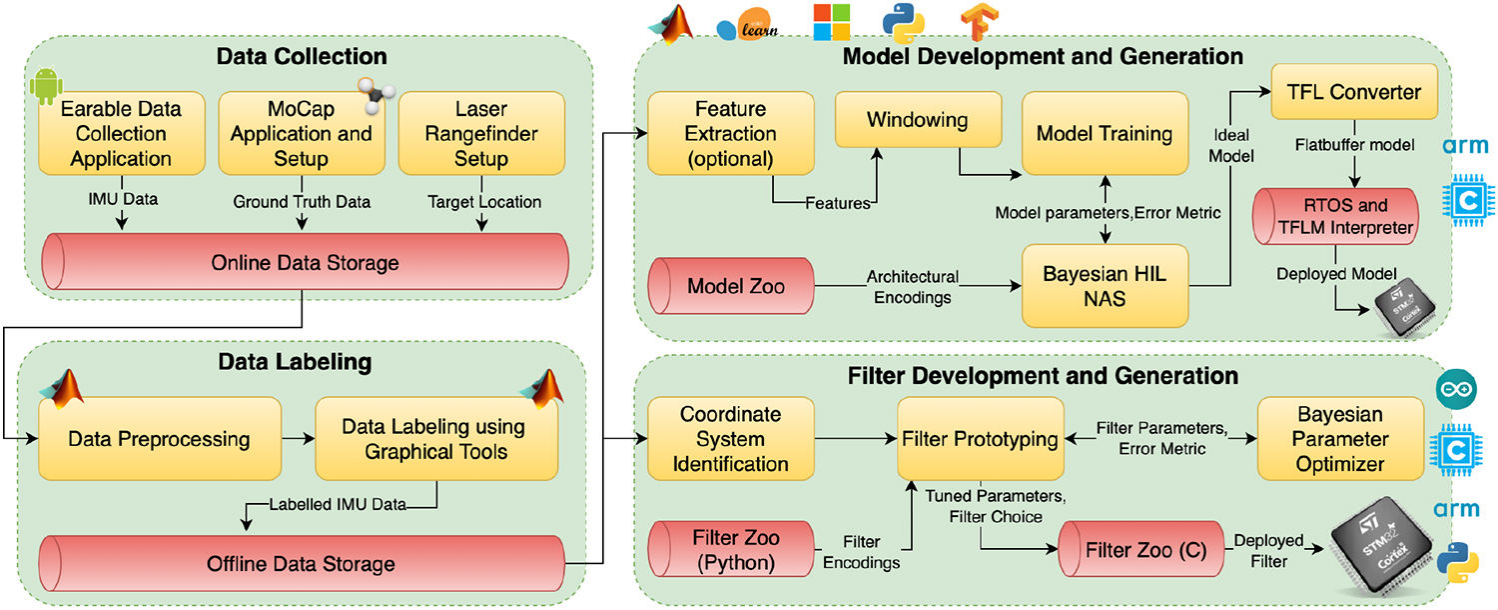
Architecture of Auritus. The first two modules take care of collecting and labeling high resolution earable data interactively. The development and generation modules allow model and filter optimization through automated HIL Bayesian NAS and optimization, respectively. Yellow boxes signify a process (e.g., transformation, optimization, etc.) and red cylinders signify stored artefacts (e.g., data, models, libraries, etc.).

To address the above challenges hindering earable research, we propose an open-source^1^ and extendable optimization toolkit called Auritus that supports an earable research life cycle from data collection to algorithmic development. To tackle earable dataset scarcity, Auritus provides access to an open-source, large-scale, IRB-approved^2^ earable dataset from 45 volunteers containing 34 distinct head-poses and 9 classes of simple activities of daily living (ADL) with 2.43 million samples. The dataset is large enough to train high-accuracy activity classifiers and head-pose filters encompassing sufficient statistical diversity. We provide both sliced sequences and continuous trajectories in the world coordinate frame. Further, Auritus is accompanied with tools (data collection and labeling components in Fig. 1) to enable similar dataset collection, pre-processing, and labeling by other researchers and application developers.

To enable the development and training of ML classifiers targeted for resource-constrained earable platforms, Auritus performs ML model training using completely automated hardware-aware neural architecture search (NAS). We achieve this optimization through tight integration of state-of-the-art (SOTA) advances in TinyML and NAS [78, 85] in the model development and generation stages as shown in Fig. 1. We develop a zoo of well-characterized pre-trained activity classification models and head-pose estimation filters using the optimization workflow in both Python and C targeted towards five different microcontroller devices. The collection contains five lightweight activity models, five conventional activity models, and four lightweight head-pose filters at various points in accuracy - model size space with performance superior to existing models and filters. In our evaluation, we achieve 98% activity detection accuracy (91% leave 1-out accuracy) with 6-13 kB models, which is 98-740× smaller and 3-6% more accurate than the state-of-the-art (SOTA). The included head-pose estimation filters have errors as low as ~5 degrees with a size of 20 kB; providing 1.6× improvement over the SOTA.

Finally, we showcase three representative applications developed using the workflow provided by Auritus, namely fall detection, spatial audio rendering, and AR interfacing. The resulting software, applications, and libraries are completely open-source, explicitly designed to facilitate replication and use by others. Overall, Auritus provides a way to allow others to develop new human movement models and filters on our dataset, collect new data, label data, and compare the results with prior work through a combination of automated scripts and human-in-the-loop. Our target communities include researchers who will benefit from our benchmark dataset by allowing them to compare their newly developed models with existing ones. We also target practitioners looking to deploy onboard human motion inference models and filters by optimizing for specific hardware platforms. Our contributions are summarized as follows:

- **End-to-End Earable Application Development Framework:** To the best of our knowledge, we are the first to provide an end-to-end learning-enabled application development framework for earables. The framework includes the first large-scale open-source human movement dataset, data collection, and labeling tools, a hardware-aware optimization toolkit, and a zoo of well-characterized human movement models and filters.
- **Hardware-in-the-loop Network Architecture Search:** Among all existing NAS frameworks developed for microcontrollers [8][27][56][57], we are the first to provide a gradient-free Bayesian NAS for microcontrollers that supports use of both proxies and communication with real hardware in real-time to receive hardware metrics. We show that existing NAS frameworks fall short due to absence of real hardware or proper search strategy in the NAS phase.
- **A Zoo of Lightweight Models and Filters:** We develop and provide 5 lightweight models, 5 conventional models, and 4 headpose filters with Auritus for activity detection and head-pose estimation. The said lightweight models and filters have not been used in conjunction with NAS for earable activity detection and head-pose tracking before.
- **Pushing the State-of-the-Art:** Our lightweight models and filters significantly reduce inference error and resource consumption than existing earable activity classifiers and head-pose filters. We also showcase three representative applications using Auritus that exploit the optimization pipeline.
- **Challenges and Limitations in TinyML:** Through Auritus, we find several unsolved technical problems characteristic not only for earables, but the overall TinyML pipeline itself.

### 1.3 Organization

The rest of the paper is organized as follows: Section 2 presents background and related work. Section 3 illustrates the data collection setup, statistical tests on participants and activities, and our semi-automated graphical data labeling tool. Section 4 details both the training and development pipeline for HIL activity detection models and filters. Afterwards, Section 5 presents extensive experimental evaluations of the developed models and filters. Section 6 provides three exemplary applications developed through Auritus. Finally, Section 7 provides concluding remarks and future directions.

## 2 BACKGROUND AND RELATED WORK

There is a serious push towards embedding sensors in commercial earbuds. Apple, Samsung, Bose, and Microsoft have already embedded directional microphone arrays, touch sensors, and accelerometers in their earbuds for speech, motion detection, and gesture sensing [19, 26]. Bragi earbuds are also capable of performing in-ear fitness tracking, gesture recognition, and passive noise reduction [73]. In this paper, we focus on human activity detection and head-pose recognition using earables that enable multiple interesting and diverse applications as listed below:

### Health Monitoring:

Gil *et al*. [32] proposed an earable device to measure cardiovascular conditions during physical exercise. The authors fuse ECG, impedance, amperometric, and potentiometric measurements with 3DoF inertial measurements to capture the electric potential around the ear. Roddiger *et al*. [80] showed that filtering, interpolation, and principal component analysis can allow earables to measure respiratory rates. Nirjon *et al*. [66] showed that it is also possible to detect heart rate using ear-worn sensors. Recently, electrodes are being used in earables to monitor sleep quality through in-ear EEG measurements [60].

### Context Detection:

Emotion regulator conversational agents have been ported to earbuds to dynamically adjust conversation style, tone, and volume in response to the wearer’s emotional, environmental, social, and activity context. The context is gathered through speech prosody, motion signals, and ambient sound [43].

### AR, MR and Tangible Interfaces with 3D Sound:

Yang *et al*. [102] illustrated the fusion of acoustic and inertial sensors from earbuds and smartphones to project 3D binaural audio based on wearer location and gazing orientation. Nasser *et al*. [65] presented an AR concept with earables to provide thermal haptic cues near the ear region, which then provides directions and notifications to visually impaired individuals. Taniguchi *et al*. [93] designed an augmented earable hands-free interface to control devices using tongue movements.

### Security, Authentication, and Speech Recognition:

Head motion signatures and inertial disturbances generated during speech can be used for standalone authentication purposes [19, 98]. This is useful to counteract voice fingerprinting attacks. In addition, the fusion of IMU and the microphone can be potentially harnessed to identify the voice in noisy environments [19].

### Dead-Reckoning:

Prakash *et al*. [74] exploited the natural filtering of noise/vibrations (associated with inertial sensor data) as inertial signals propagate from lower-body to the ear canals to develop a subject, and pattern-independent step counting reflex model (called STEAR) using dynamic time warp (DTW) algorithm. Ahuja *et al*. [3] showed that it is possible to perform on-board dead-reckoning on earables using magnetometer-based heading estimation and a belief-based step-counting algorithm from an earable IMU. The directional beamforming microphones in earables can also be used to perform ultrasonic echo-localization for generating indoor maps [72].

### 2.1 Activity Detection and Head-Tracking Using Earables

To better facilitate earable computing applications, it is necessary to realize more accurate activity detection. Researchers [5, 38, 74, 75] have proposed several ML classifiers for activity detection using earables, summarized in Table 1. The location of earables enables the natural filtering of noise and vibrations by the upper body, providing the potential for high accuracy and multi-granular activity detection with earables [19, 74, 75]. In fact, Atallah *et al*. [5] validated the use of a 3DoF earable sensor for gait monitoring by correlating acceleration features with gait parameters from a piezoelectric force-plate instrumented treadmill. Existing techniques have used convolutional neural network (CNN), k-nearest neighbors (kNN), random forests (RF), and k-means clustering for multiscale activity detection. These include distinguishing between head-mouth (verbal) and full-body mechanics (non-verbal) [38, 51], exercise activity detection [75, 77], facial expression detection [54, 91, 96], and activity-level detectors [66]. However, existing work completely ignores the compute constraints of earables. As shown in Table 1, these algorithms are not deployable on embedded earable platforms due to their model size requiring high RAM usage or having feature extraction overhead. In contrast, Auritus uses hardware-aware NAS to train ML classifiers targeted towards microcontroller class processors typically used in earable platforms. Our trained models are directly transferred to the earable platforms to benchmark their performance within the computation limits of earables.

With the advent of virtual reality (VR) and AR applications, the innate challenges of head-tracking using inertial sensors have also been explored. Existing works usually perform simple gyroscope integration [102], often fusing data from two earables with tilt/yaw correction and predictive/positional tracking [28] from tertiary head-tracking devices (e.g., Oculus Rift). However, gyroscopes suffer from time-varying bias due to bias instability and angular random walk, leading the integral to drift over time [48]. To enable long-term head motion tracking, Auritus provides lightweight yet accurate head-tracking filters that can account for sensor errors statistically or heuristically. The filter parameters are optimized using Bayesian optimization (BO).

### 2.2 Neural Architecture Search

Several NAS frameworks have been proposed for microcontroller-class devices. SpArSe [27] treats NAS as a gradient-driven multi-objective BO problem, treating hardware attributes via proxies and coupling pruning with NAS. MicroNets [8] uses a quantization-aware gradient-driven approach to optimize task-aware DNN backbones. MCUNet [57] tailors Once-for-All (OFA) NAS [12] for microcontrollers, using a two-stage evolutionary NAS to train a single OFA network in an optimized search space for a broad spectrum of target hardware. Adopting MCUNet is a challenge as it uses a custom inference engine and its latency/resource measurements rely on a closed-source software stack. In Auritus, we perform hardware-aware NAS using multi-objective BO, where the acquisition function is optimized using Monte Carlo sampling.

We adopt BO due to the following reasons: (i) BO provides a state-of-the-art approach to optimize expensive objective functions in a few evaluations [87], (ii) BO allows explicit inclusion of non-gradient-friendly constraints of the model size and accuracy tradeoffs during the training process [27]. The choice of Monte Carlo sampling instead of the gradient-driven approach of SpArSe [27] is based on the fact that neural architecture search space consists of categorical variables where the sampling approach evaluates the acquisition function only at valid configurations only [31, 86]. Auritus includes the hardware-aware training where the resource utilization of a model is computed at runtime by its real deployment on the target hardware, instead of just using proxies as done by SpArSe [27]. Our evaluation shows that proxies are only approximations of the real hardware constraints, which are noisy for extremely resource-constrained devices. In addition, none of the NAS frameworks can optimize energy and Tensorflow Lite Micro arena size in real-time, as they do not use real-hardware during the NAS.

## 3. DATA COLLECTION AND LABELING PIPELINE

To address the challenge of open source data scarcity for earable, Auritus provides a pipeline specifically designed to ease data collection, pre-processing, and labeling of earables. In this section, we introduce the experimental testbed used to collect human movement data using earables (Section 3.1) and describe statistical tests on the collected data, participants, and activities (Section 3.2) to verify data quality. To ease the data labeling effort, Auritus also incorporates a graphical data-labeling tool (Section 3.3).

### 3.1 Data Collection Setup

**Fig. 2.**
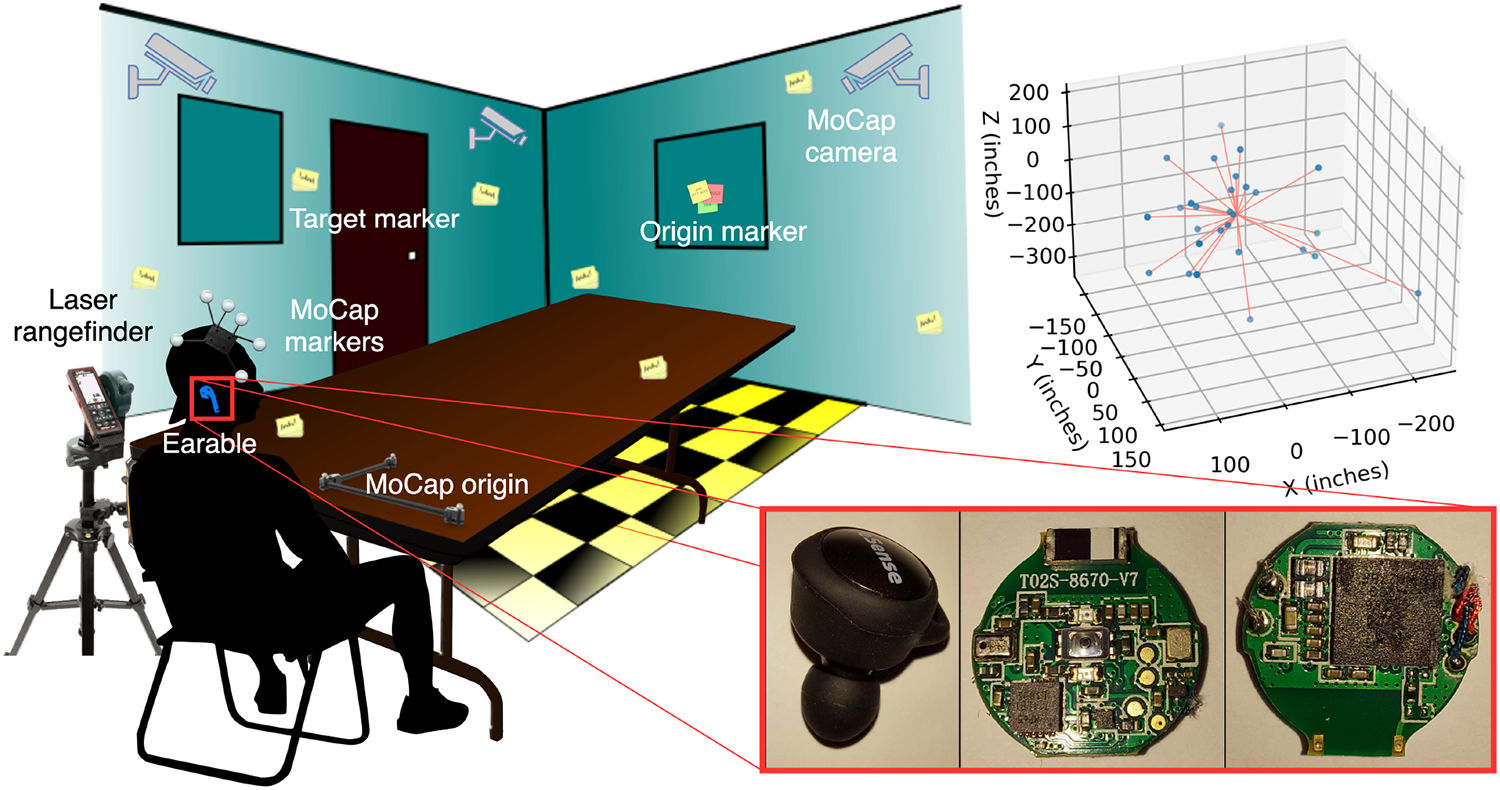
Experimental setup for head-pose and ground truth data collection, with the positions (in inches) of target markers characterized in Cartesian coordinates w.r.t. origin marker.

For data logging, we used the popular eSense^3^ earable device from Nokia Bell Labs [45, 46], shown in Fig. 2. We used the built-in Butterworth filter with the cutoff set to 5 Hz to reject high-frequency noise beyond motion parameters. We used BLE advertisement and connection intervals of 45-55 mS and 20-30 mS, respectively. The 6-channel inertial data was broadcasted at ~100 Hz to an in-house smartphone application we developed using eSense Android middleware backend [45].

The experimental testbed for head-pose data collection is also illustrated in Fig. 2. For sub-mm resolution ground truth collection, the participants wore a hat with OptiTrack Prime 17W^4^ MoCap infrared markers [29] mounted in a rigid body configuration. The motion data of the head and the marker visual cues were tracked using Motive:Tracker ^5^ [6] and screen recorder applications, respectively. To synchronize the discrete data management elements, we harmonized the local system clocks to the Network Time Protocol (NTP) [62] and graphically identifiable calibration nods performed before the data collection phase. The testbed contained 27 target markers, one of which is an origin marker. A participant is supposed to move the head to and from an origin marker to target markers. To characterize the position of each marked point in the 3D testbed, we used a Leica Disto X3 laser rangefinder^6^ and a digital compass to obtain the distance, azimuth, and elevation angles of the targets from the participant’s position. To log activity data, we asked participants to perform nine common ADL [83] after providing them sample cues. Each participant performed calibration nods in between each activity to signify the start and endpoints for each ADL. We ensured that there were no path obstructions or distractions during activity data collection and restarted the process when the earable became loose or any unforeseen circumstances arose.

### 3.2 Participants and Activities

For the head-pose dataset, we collected 34 distinct head-poses from the 27 targets per participant, obtaining ~2.43 million inertial samples in total. We considered two common types of head movements. The simple class included movements of the head from the origin marker to a target marker and back to the origin marker. We collected 411,103 such samples (after preprocessing). The more complex class involved movements of the head from the origin marker to a target marker 1 (phase A), target marker 1 to a target marker 2 (phase B), and target marker 2 to the origin marker (phase C). We collected 1,068,211 samples for the complex head movements (after preprocessing). For the activity dataset, nine classes of actions were recorded, illustrated in Table 2. The length of time for each activity varied for each participant, but on average, each activity IMU trace for each participant was around 23-25 seconds. A total of 958,182 inertial samples were recorded (after preprocessing). We collected data from 45 participants (29M, 16F) in total. The sampling rate at which the accelerometer and gyroscope data was collected was set to 100 Hz. For each participant, we calibrated the gyroscope and the accelerometer three times (once before collecting simple head movements, once before collecting complex head movements, and once before collecting activity recognition data) to remove gyroscope static bias drift and set up the accelerometer gain factors and accelerometer biases using static calibration techniques described in [20] and [100]. For gyroscope calibration, we placed the earable on a flat table in a static position, sampled the gyroscope for 10 seconds, and averaged the readings to get the static bias value which we subtracted from subsequent gyroscope readings. For accelerometer calibration, we placed the earable in six different stationary tilt angles and used the iterative method in [100] to estimate the gain factors, *G*_*i*_ and biases, *B*_*i*_ for each axis. The true acceleration in each axis is then given by: *A*_*i*_ = (*S*_*i*_ − *B*_*i*_)/*G*_*i*_, where *S*_*i*_ is the raw accelerometer readings for each axis.

To ensure sufficient statistical diversity in physiological parameters without bias, the Kolmogorov-Smirnov test [40] was performed on age, weight, height, and ear height from the origin. Table 3 summarizes the normality test results. No bias was observed in age, height, weight, and ear height from the origin, and at the 0.05 level, all parameters were significantly drawn from a normally distributed population.

**Fig. 3.**
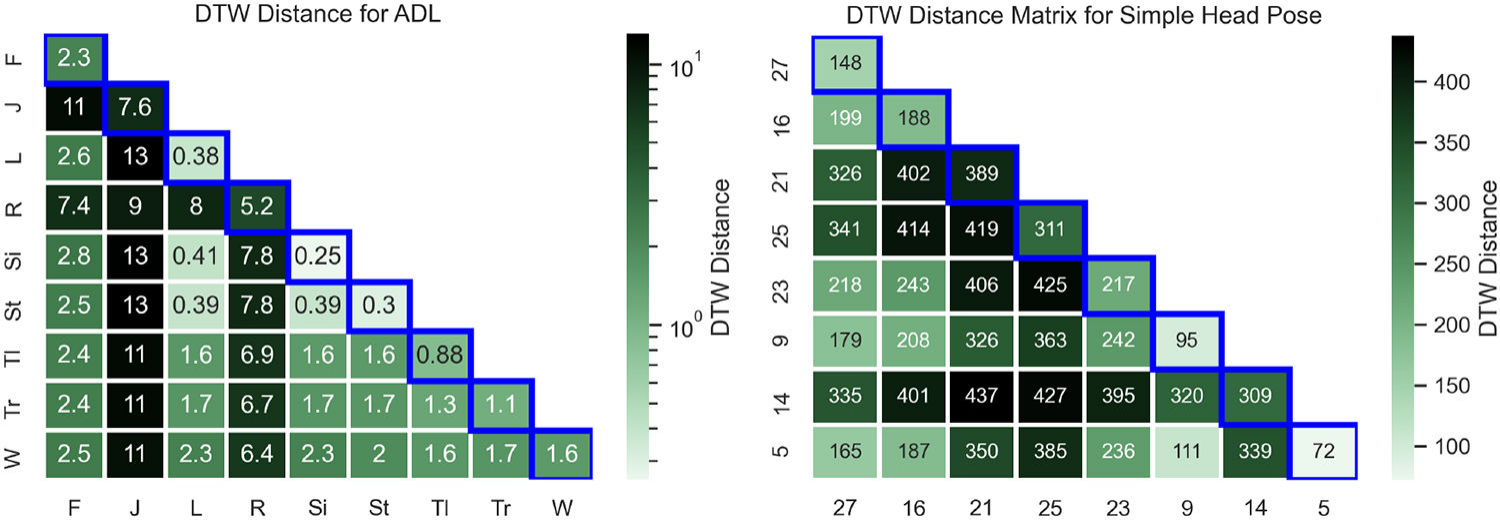
Heatmap of average DTW distance across all activity classes and selected simple head movements. The distance should be small among inertial traces of same class (marked with blue bounding box) and large for different classes.

Fig. 3 illustrates the heatmap of the average dynamic time warping (DTW) distances among the motion traces of the same class versus different classes. The DTW distance provides a measure for the similarity between two temporal sequences with different speeds [9]. Intuitively, inertial traces belonging to the same class should have a small DTW distance, and signals belonging to different classes should have a large DTW distance. We applied Kruskal-Wallis ANOVA on the average DTW distance across 10 random accelerometer vector sum snapshots ($\sqrt{A_{x}^{2}+A_{y}^{2}+A_{z}^{2}}$) from different participants, with each snapshot being 400 samples in length. The results are illustrated in Table 4. At the 0.05 level, the distributions pertaining to the DTW distances from the same class and different classes are significantly different, indicating the presence of well-separated clusters for each ADL in low-dimensional latent space learnable by ML algorithms. The same test was applied to selected simple head movements (varying sample count) from different participants belonging to eight random target markers. The inertial traces consisted of gyroscope sum snapshots (*ω*_*x*_ + *ω*_*y*_ + *ω*_*z*_), and the same statistical inference was observed for head movements.

### 3.3 Graphical Data Labeling Tool

To ease labeling time-series data collected in continuous chunks, we designed a graphical inertial data labeling tool to allow head-pose and activity data annotation using a graphical-user-interface (GUI). Using the aid of the ground truth videos from Motive:Tracker, the application developer selects points directly on the plot signifying the start and endpoints of calibration nods and head movements. A single head rotation on the gyroscope-time plot essentially consists of a triangular/bell-curved shape peak, with the rate of change of the angular velocity proportional to the head motion velocity (faster = thinner and taller peak, slower = thicker and smaller peak). After specifying all the endpoints and making any numerical adjustments to the data, the developer exports the endpoints to the GUI workspace and runs a script to perform automatic segmentation and labeling based on the endpoints. Three such scripts are provided, one each for activity, simple head-pose, and complex head-pose. The developer only needs to input the volunteer number and labels to the scripts.

## 4 DEVELOPMENT OF MODELS AND FILTERS

Although ML classifiers for activity detection are well explored in the domain of smartphones and smartwatches [37, 81, 99], their direct transfer to the resource-constrained domain of earable platforms is not feasible. Auritus incorporates the ML model training in combination with completely automated hardware-aware NAS. The NAS is designed to ensure classifier inference is directly possible on edge in real-time within the available flash and RAM constraints of the target device. Given different inputs and different requirements from the application developer, the optimization workflow produce different model implementations automatically. The system can either select to optimize a specific model or give a model based on developer requirements. Further, to provide superior head-pose tracking, Auritus includes a set of filters with different computation complexity. We provide the models and filters in Python and C for real-time application on embedded hardware via Mbed real-time operating system (RTOS) and TensorFlow Lite Micro (TFLM) backend. In this section, we outline the pipeline for training and developing activity classifiers from the model and filter zoo on real hardware using Bayesian HIL NAS and lightweight model architectures (Section 4.1). Next, we delineate the activity classifier implementation details by discussing feature extraction, windowing, dataset splits, design space, and hardware/software details (Section 4.2). Last, we discuss the generation of head-pose estimation filters (Section 4.3)

### 4.1 Hardware-Aware Lightweight Model Generation

The memory and compute capability of TinyML devices are significantly smaller than cloud or even mobile devices. For example, an Arduino BLE33 has only 320 KB of SRAM and 1 MB of flash, compared to 4 GB of RAM and 64 GB of storage on a smartphone. A GPU can have 16 GB of memory on a workstation with secondary storage in the order of terabytes. Thus. optimizing larger models for smaller devices directly using techniques such as dimension reduction, pruning, quantization, and model compression alone are insufficient to mitigate the loss of accuracy [12, 57]. Moreover, the type of ML operators supported by such devices is limited by the processor architecture and the runtime interpreter. For example, vanilla recurrent neural network (RNN) operators are not widely supported by off-the-shelf TinyML software frameworks [8]. As a result, the design goals of models and the ML operator space should be optimized through the integration of novel lightweight model design paradigms and target hardware specifications in order to strike an equilibrium between accuracy and efficiency [27, 49, 57].

#### 4.1.1 Hardware-Aware Bayesian NAS.

To find the ideal activity detection model candidate from a backbone deep neural network (DNN) search space for limited flash, RAM, and latency requirements, we model the search as a parallelizable black-box BO problem. The search space Ω consists of neural network weights *w*, hyperparameters *θ*, network structure denoted as a directed acyclic graph (DAG) *g* with edges *E* and vertices *V* representing activation maps, and common ML operations *υ* (e.g., convolution, batch normalization, pooling, etc.) which act on *V*. The goal is to find a DNN that maximizes the hardware SRAM and flash usage within the device capabilities while minimizing latency and classification error on the validation set.

(1) $$f_{\text{opt}}=\lambda_{1}f_{\text{error}}(\Omega )+\lambda_{2}f_{\text{flash}}(\Omega )+\lambda_{3}f_{\text{SRAM}}(\Omega )+\lambda_{4}f_{\text{latency}}(\Omega )$$

where

(2) $$f_{\text{error}}(\Omega )={𝓛}_{\text{validation}}(\Omega ),\Omega =\{\{V,E\},w,\theta ,v\}$$

(3) fflash(Ω)={−‖hFB(w,{V,E})‖0flashmax∨−HIL informationflashmax∞,fflash(Ω)>flashmax}

(4) fSRAM(Ω)={−maxl∈[1,L]{‖xl‖0+‖al‖0}SRAMmax∨−HIL informationSRAMmax∞,fSRAM(Ω)>SRAMmax}

(5) $$f_{\text{latency}}(\Omega )=\frac{\text{FLOPS}}{{\text{FLOPS}}_{\text{target FLOPS}}}\;\vee \;\frac{\text{HIL information}}{{\text{Latency}}_{\text{target latency}}}$$

The objective function *f*_opt_ can be thought of as seeking a pareto-optimal configuration of parameters Ω* under competing objectives [27], such that:

(6) $$f_{k}(\Omega^{*})<=f_{k}(\Omega )\;\;\forall k,\Omega \;\;\wedge \;\exists j\;:f_{j}(\Omega^{*})<f_{j}(\Omega )\;\;\forall \Omega \neq \Omega^{*}$$

**Fig. 4.**
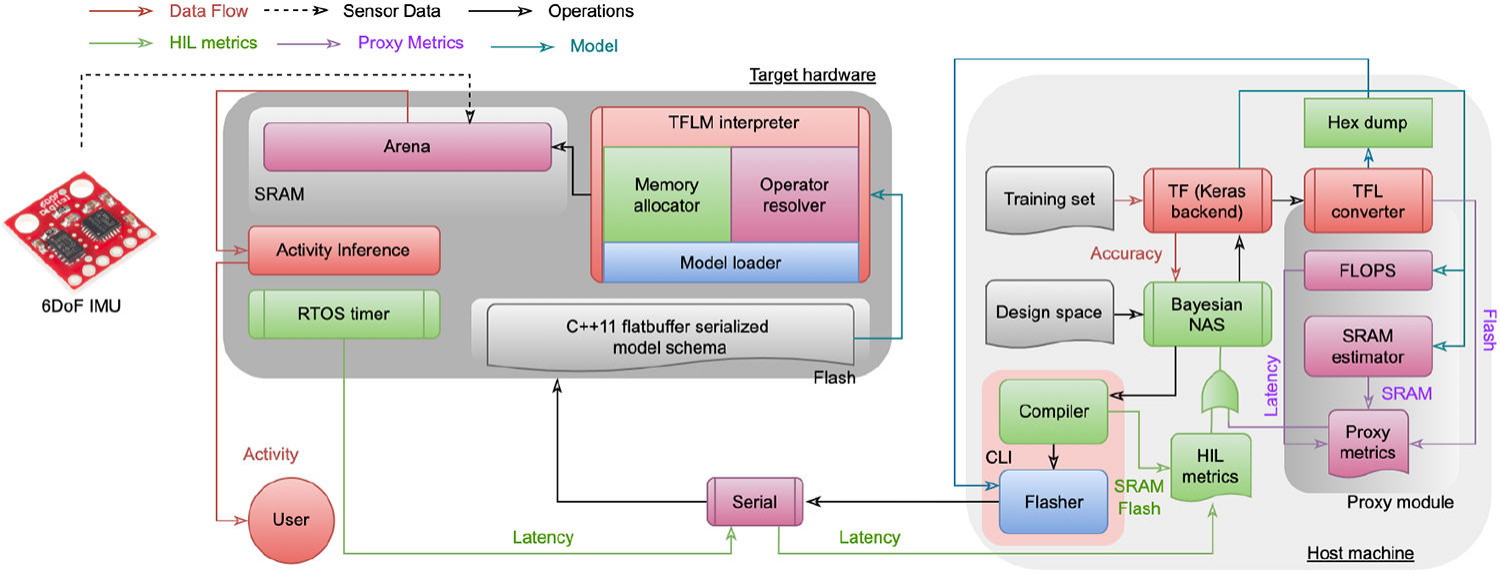
HIL model optimization for earable activity detection using Bayesian NAS. The system supports both the use of proxy and real hardware to get hardware constraint estimates.

First, validation accuracy serves as a proxy for the error characteristics *f*_error_ (Ω) of the model. Secondly, the size of the serialized flatbuffer model schema *h*_FB_ (·) [21] generated by TFL acts as a proxy for flash usage when real-hardware is absent. Thirdly, off-the-shelf tools such as TFLM store network weights, quantization parameters, and network graphs on flash. These tools use a predefined portion of the SRAM called the arena to store intermediate activation maps and tensors, persistent buffer, and TFLM runtime interpreter parameters. We use this standard RAM usage model as a proxy for SRAM usage *f*_SRAM_(Ω) [27]. Lastly, since model latency is linearly proportional to the OPS count for a variety of convolutional models for TinyML devices, we use FLOPS or OPS as a proxy for runtime latency [8]. When real hardware is available, we obtain the SRAM, flash, and latency parameters directly via the serial interface from the target compiler and RTOS, illustrated in Fig. 4. We normalize all the hardware parameters by device capacity or target metrics.

We use Gaussian process 𝓖𝓟 as the surrogate model to approximate *f*_opt_, which allows priors on the distribution of moments to propagate forward as the search progresses. In addition, the domain of random scalarizations *λ* can be specified by the developer to guide the parallel search acquisition functions (hallucination or K-means clustering) into the promising Pareto-optimal regions of the gradient plane. The acquisition function decides the next set of Ω_*n*_ to sample from the design space using Bayesian Upper-Confidence Bounds (UCB), which balances exploration and exploitation [86]. Apart from speeding up the NAS, parallel search ensures that NAS is not being performed on network morphs early on (exploitation) and information gain is maximized in the search process (exploration), yielding a stage-wise "coarse-to-fine" search space.


(7) $$\hat{f}(\Omega )∼𝓖𝓟(\mu (\Omega ),k(\Omega ,\Omega^{\prime }))$$

(8) $$\Omega_{t}=\text{arg}\;\max_{\Omega }(\mu_{t-1}(\Omega )+\beta^{0.5}\sigma_{t-1}(\Omega ))$$

Note that while minimizing the latency and classification error within the hardware SRAM and flash bounds of the device should generate classifiers that perform reasonably well in theory, we observed that without *f*_flash_(Ω) and *f*_SRAM_(Ω) in *f*_opt_, the NAS program generates models that do not fully exploit the device capabilities and produces small models that may be 2 – 5% less accurate than larger models. *f*_flash_(Ω) and *f*_SRAM_(Ω) act as regularizers in *f*_opt_, penalizing the NAS program for picking small models, while also promoting the generation of a fine-grained surrogate model. Note that all SOTA NAS frameworks for microcontrollers [8][27][56][57] use a formulation similar to *f*_opt_.

#### 4.1.2 Conventional Activity Classifiers.

We included five conventional ML activity classifiers from literature, namely bagged trees [83], AdaBoost [52], coarse decision tree (DT) [2], support vector machine (SVM) [4][83], and multilayer perceptron (MLP) [82] in the model zoo to compare against lightweight activity classifiers.

- **Bagged Trees**: Bootstrap aggregation combines several decision trees trained on bootstrap samples to form an ensemble classifier, using majority voting to provide the final label [83].
- **AdaBoost**: Combines weak decision stumps to form an ensemble classifier in a weighted form depending on misclassified points [52].
- **Coarse DT**: Graph of decisions where each node makes binary decisions based on values of the input activation and predefined rules, optimized through splitting and pruning [2].
- **SVM**: An SVM finds a linear decision hyperplane in the feature space whose margin is maximum from the support vectors (cleanly split examples) of two classes, using kernels to project data into a linearly separable manifold [4][83].
- **MLP**: A 2-layer fully-connected feedforward neural network with sigmoid hidden neurons, trained using scaled conjugate gradient backpropagation with cross entropy loss [82].

#### 4.1.3 Lightweight Activity Classifiers.

To enable real-time activity classification on resource-constrained devices through our HIL model optimization tool, we designed and included several lightweight classifiers suitable for onboard activity inference in the model and filter zoo. We implemented temporal convolutional network (TCN) [53, 94], fast gated RNN [50], fast RNN [50], Bonsai [49] and ProtoNN [35] models for lightweight yet accurate activity detection. These models use several design techniques to reduce model size and latency while maintaining performance on-par with conventional ML and deep learning (DL) algorithms for time-series processing:

- **Temporal Convolution**: Without explosion of parameter, memory footprint, layer count, or overfitting, TCN kernels allow the network to discover the global context in long inertial sequences while maintaining input resolution and coverage. In TCN, the convolution operation has three desirable properties:
  - *Causality*: The output of the operator at the current timestep *t* depends only on the current and past inputs but not future inputs. This ensures temporal ordering of the input sequences without requiring recurrent connections. The ordering is maintained via weight sharing among the input chunks.
  - *Dilated Convolution*: The receptive field *F*_*i*_ of each unit in the *i*th layer in a TCN dilated causal kernel of size *k* × *k* with dilation factor *l* is given by:
(9) $$F_{i,\text{TCN}}=F_{i-1}+(k_{l}-1)\times l,F_{0}=1$$
    *F*_*i*,TCN_ is larger than *F*_*i*,CNN_, which is *i* × (*k* − 1) + *k*. When dilated CNN are stacked on top of each other, the dilation factor increases exponentially, increasing model capacity and receptive field size with fewer layers and parameter count over vanilla CNN or RNN
  - *Residual Blocks:* Two stacks of dilated causal convolution layers, *f* and *g*, are fused through gated residual blocks **z** for expressive yet bounded non-linearity, complex interactions and temporal correlation modeling in the input sequence:
(10) $$z=\tanh(W_{f,k}*x)⊙\sigma (W_{g,k}*x)$$
    where **W** are the weights in each layer, *σ* is the sigmoid function and **x** is the input.
- **Stabilized RNN with LSQ Matrices**: Vanilla RNN, albeit lightweight, suffer from exploding and vanishing gradient problem (EVGP) for long temporal sequences. Existing solutions to EVGP (e.g., gated RNN (long short-term memory (LSTM) and unitary RNN) come at the cost of either accuracy loss or increased memory and latency overhead. Fast RNN [50] solves EVGP by adding a weighted residual connection with two scalars (*α*, *β*) to generate well-conditioned gradients:
(11) $${\tilde{h}}_{t}=\sigma (Wx_{t}+Uh_{t-1}+b),\;h_{t}=\alpha {\tilde{h}}_{t}+\beta h_{t-1}$$
  where 0 ≤ *α* ≪ 1, *β* ≈ 1 − *α*, *β* ≤ 1, *σ* is a non-linear activation function, **W** and **U** are RNN matrices, **b** is bias vector, **h** is the hidden state and **x** is the input. By varying *α* and *β*, we can control the update extent of **h**_***t***_ based on **x**_***t***_. Fast GRNN [50] then converts this residual connection to a gate while enforcing **W** and **U** to be low-rank, sparse and quantized (LSQ):
(12) $${\tilde{h}}_{t}=\tanh(W^{\prime }x_{t}+U^{\prime }h_{t-1}+b_{h})$$
(13) $$h_{t}=(\zeta (1-z_{t})+v)⊙{\tilde{h}}_{t}+z_{t}⊙h_{t-1},\;z_{t}=\sigma (W^{\prime }x_{t}+U^{\prime }h_{t-1}+b_{z})$$
(14) $$W^{\prime }=W^{1}(W^{2}{)}^{T},\;U^{\prime }=U^{1}(U^{2}{)}^{T}$$
  where, *ζ* ≥ 0, *υ* ≤ 1. Fast GRNN, thus, is able to provide the capabilities of gated RNN without the associated compute overhead.
- **Sparse Low-Dimensional Projection**: Bonsai [49] is a shallow and sparse DT with non-linear activations, making inferences on data projected in low-dimensional space called prototypes. Similarly, ProtoNN [35] is a lightweight k-nearest neighbor (kNN) classifier designed to operate on prototypes. The sparse projection matrix is learned using stochastic gradient descent and iterative hard thresholding. Sparsely projecting high-dimensional feature space onto a low-dimensional linear manifold reduces parameter count for Bonsai and ProtoNN, allowing them to be computationally efficient.

### 4.2 Activity Classifier Implementation Specifics

In this sub-section, we provide details on the implementation of the activity classification training pipeline, including feature extraction, windowing, dataset splits, design space optimization, and specifications of target hardware and host machine for training.

#### 4.2.1 Feature Computation and Windowing.

For activity classification using conventional ML algorithms, 241 spatial features were extracted from our dataset with varying sliding window sizes (1, 3, 5, and 10 seconds) and stride of 0.5 seconds, shown in Table 5. Each feature (except the time window) was applied separately to 3 accelerometer and 3 gyroscope channels (180 features). Each feature (except the time window) was also applied to the vector sum of accelerometer and gyroscope channels (60 features). We included time window as a feature to account for sampling rate jitter and missing data in the dataset [84]. For Bonsai and ProtoNN, we apply five lightweight features from the 241 features on the accelerometer and gyroscope vector sums, namely maxima, minima, range, variance, and standard deviation, totaling 10 features. A sliding window of varying size (1,2,3 and 5 seconds) with a stride length of 0.5 seconds were chosen for Bonsai, ProtoNN, Fast RNN, Fast GRNN, and TCN. We do not extract any features for Fast RNN, Fast GRNN, and TCN and feed raw windowed inertial samples to the three classifiers. For all classifiers, no normalization or standardization was applied to the raw data.

#### 4.2.2 Hardware and Software Specifications.

All models were trained on a host machine with 256 GB RAM, 2× 24 GB Nvidia GeForce RTX 3090, and 3.7 GHz AMD Ryzen Threadripper 3970X 32-core CPU. For benchmarking HIL NAS, we use three real ARM Cortex-M target boards and two virtual hardware models (proxies) with varying resource constraints. The processors run Mbed RTOS and TFLM interpreter on-board. To communicate with the target hardware via system commands from the host machine, we used the Mbed command-line interface (CLI). The target hardware specifications are outlined in Table 6:

All the conventional ML models were implemented in MATLAB and later converted to C optimized for Cortex-M processors. All lightweight models were implemented in Jupyter notebook (Python), using Keras and Microsoft EdgeML via a Tensorflow and TFLM [21] backend. The TCN, ProtoNN, and Bonsai models were converted to flatbuffer model schema [21] using TFL and the other models were converted to C-compatible formats for further benchmarking. All models used cross-entropy loss except Bonsai, which used multi-class hinge loss.

#### 4.2.3 Dataset Splits.

We split the dataset in three different ways for our evaluation:

- **Split with no unseen participants:** In this split, there are no unseen participants, i.e. data from all participants are present in the training set. This was used to report **test accuracy**. We used holdout validation (train: validation: test) ratios of 80:10:10 and 70:15:15 for classical models and MLP, respectively. For the lightweight models, we used holdout validation of 80:10:10 for the TCN and 90:0:10 for Bonsai, ProtoNN, FastRNN, and FastGRNN. Except for MLP, we ensured that the dataset splits are the same for all classifiers for a fair comparison.^7^
- **Split with leave-1 out:** The data is split per user, such that the models are trained on data from 44 participants, and tested on data from a participant not present in the training set. This was used to report **leave-1 out test accurac**y. We performed a 10-way cross-validation, choosing a random participant each time to be left out of the training set while the model is trained on other 44 participants. We then average the leave-1 out accuracy of the 10 models. The train: validation ratio was 90:10 for the data from the 44 participants.
- **Split with leave-n out:** The data is again split per user, however, the number of participants left out now varies. This was used to perform **leave-n out cross-validation studies**. The train: validation ratio was 90:10 for the data from the participants present in the training set.

#### 4.2.4 Design Space Optimization.

For the conventional classifiers, we used a variation of the BO pipeline we showed earlier for hyperparameter tuning. We used 80 iterations for the candidate models in the search space with the expected improvement per epoch as the acquisition function to select the most optimal hyperparameter for each model. We did not include hardware constraints in *f*_opt_ for conventional ML models but aimed to maximize the test accuracy of the conventional activity detection models. For the five lightweight classifiers, we used 50 iterations for the candidate models in the search space. We incorporate hardware constraints only for the TCN in *f*_opt_, as we observed Bonsai, ProtoNN, Fast RNN, and Fast GRNN to be resource-efficient without requiring explicit hardware-aware optimization by design. Table 7 lists the architectural search space for all 10 models in the model and filter zoo, as well as support for HIL optimization. We fixed some of the parameters of each model to default or well-known values and excluded them from the search space.

### 4.3 Head-pose Filters

For real-time head-pose estimation, we fed the raw, unprocessed head-pose streams to analytical orientation estimation algorithms. We include four filters in the model and filter zoo for head tracking. To optimize the filter parameters for root-mean-squared error (RMSE) minimization, we use BO.

- **Complementary Filter**: The complementary filter [48] acts as a low-pass filter for accelerometers to mitigate high-frequency Gaussian noise, and a high-pass filter for gyroscopes to counteract time-varying drift, thereby amplifying the strengths and attenuating the weakness of each sensor in the IMU for attitude estimation. The filter simply integrates the gyroscope readings to get the 3D attitude from the gyroscope and takes linear accelerometer components in the appropriate direction to get the 2D attitude. The only tunable parameter in the filter is *β*, which weighs the contribution of accelerometer and gyroscope attitude. While the filter is simple and lightweight, it does not account for statistical treatment of drift and noise, leading to quick orientation drift. The filter also suffers from gimbal lock due to operation in the Euler domain and hence does not perform optimally for fast movements.
- **Mahony Filter**: The Mahony filter [24] solves the gimbal lock problem by operating in the quaternion domain. It also reduces the attitude drift caused by gyroscope bias by adjusting gyroscope error using accelerometer readings through proportional-integral compensation without significant markup in computation time. The two tunable parameters are *K*_*p*_ and *K*_*I*_. *K*_*I*_ mitigates the steady-state error in orientation estimation, while *K*_*p*_ reduces the rise time to the actual orientation estimate produced from accelerometer readings.
- **Madgwick Filter**: The Madgwick filter [59] improves attitude estimation error upon the Mahony filter by incorporating accelerometer attitude increment in the orientation estimation formula. The filter performs one-step gradient descent to get the optimal attitude increment from accelerometer readings. The filter is computationally inexpensive (109 scalar operations), works well for low-sampling rate IMU, and includes pre-calibration steps. The only tunable parameter in the filter is *β*, which serves the same purpose as *β* in the complementary filter.
- **Indirect Extended Kalman Filter** (IEKF): The KF is an iterative optimal state estimation algorithm (from fusing consecutive samples of single or multiple noisy indirect modalities) under Gaussian variations [41]. It is composed of prediction (process or transition or time update) and correction (measurement update). KF is a subset of Bayes filter with Gaussian prior, linear process and measurement model with Gaussian noise and satisfying Markov property, with the goal of maximizing posterior probability EKF can deal with globally non-linear system dynamics via Taylor series and Jacobians. It linearizes the non-linear process model locally about the running state mean [10]. Instead of modeling the attitude directly, IEKF models the error in attitude estimate. We use the gravity estimation from the gyroscope and accelerometer orientation as the error model, and update the actual attitude by multiplying the errors with the head pose. While IEKF yields the most accurate head-pose estimate, it is the most computationally expensive among the four filters.

## 5 ALGORITHMIC EVALUATION, COMPARISON AND DISCUSSION

In this section, we illustrate the experimental results related to the performance of our hardware-aware optimization framework, trained models, and filter zoo on our dataset. We also compare proposed models and filters with the SOTA in earable activity detection and head pose estimation. For our proposed activity detection models, we carried out activity detection on all the 9 activities reported in Table 2.

### 5.1 Activity Detection Model Size and Accuracy

Table 8 showcases the best performance of conventional ML activity classifiers in terms of test accuracy (no unseen participants), average leave 1-out test accuracy, and model size on the entire dataset. The hyperparameters stated were the most optimal found by BO. From Table 8, we can see that the test accuracy of classifiers ranges from 98.5-100%, while the leave-1 out test accuracy ranges from 81.3-91%. Even though bagged trees had the highest test accuracy among all classifiers, SVM generalized the best overall on unseen participants. However, the SVM model was also 4× larger than the bagged ensemble model. MLP had the lowest model size of 418 kB among all conventional classifiers, while AdaBoost was 195× larger (largest model among all) but ~ 1% less accurate than MLP in terms of leave-1 out test accuracy.

Table 9 illustrates the accuracy (test accuracy (no unseen participants) and average leave-1 out test accuracy), RAM usage, flash usage, FLOPS, and energy consumption of lightweight classifiers on the entire dataset. Excluding Bonsai and ProtoNN, none of the classifiers require feature extraction. For the TCN, we showcase five models targeted towards five different hardware classes (specified in parenthesis in Table 9), optimized via our HIL Bayesian NAS pipeline. To showcase energy usage for lightweight classifiers, we ran the industry-standard EEMBC EnergyRunner benchmark [7] for TCN, FastGRNN, and FastRNN running on ARM Cortex M4 processors, while using a widely used power monitor^8^ to log power usage of Bonsai and ProtoNN running on ARM Cortex-A processors. Our HIL NAS adapts the TCN model to achieve better accuracy with an increase in computing resources. The highest test accuracy of 98.3% was obtained by FastRNN, which also had the smallest model size of 6.04 kB among all models. However, FastGRNN achieved the highest leave-1 out accuracy of 91%, requiring only 7.08 kB more flash than FastRNN while being 4.3% more accurate. Observe that the largest model in Table 9 is 5.6× smaller than the smallest model in Table 8. Furthermore, FastGRNN and SVM provide the same leave-1 out test accuracy, but the former is 1700× smaller than the latter without requiring any feature extraction overhead. FastGRNN also has the lowest energy usage of 41 mW among all lightweight classifiers, which is 9mW less than the industry standard recommended power consumption^9^ for TinyML classifiers [7] We can make several high-level inferences from Table 8 and Table 9:

- The relationship between model accuracy and model size is non-linear, i.e., models with more parameters necessarily do not yield higher accuracies. With appropriate architectural encodings, it is possible to achieve better accuracy with smaller models. Han *et al*. [36] showed that only a small number of weights/parameters contribute to the model performance. Thus, we can further reduce the model size shown in Table 8 without losing accuracy significantly. The improvement is reflected through intelligent and lightweight model architectural formulations shown in Table 9.
- Lightweight classifiers are less robust to domain shifts than conventional classifiers, as evident from the leave 1-out test accuracies. This is because the lightweight classifiers do not have enough redundant weights or parameters to model globally significant attributes that may be common across all users, but instead overfit on the participant-specific characteristics in the temporal sequences, sacrificing generalizability over accuracy.
- Energy usage of the lightweight classifiers depend on the underlying hardware on which the energy benchmarks are being run, as well as runtime interpreter and RTOS being used. For example, the energy consumption of TCN ranges from 50-418 mW depending on the hardware platform. The L-series STM32 boards are branded as ultra-low-power, while the F-series STM32 boards are high-performance ^10^. Thus, the same classifier implemented on different hardware can yield different energy consumption, evident from FastRNN and FastGRNN’s implementation on two STM32 boards. In addition, Raspbian RTOS and TensorFlow Lite interpreter consume more power to run the same model over Mbed/Arduino RTOS and Tensorflow Lite Micro interpreter.

**Fig. 6.**
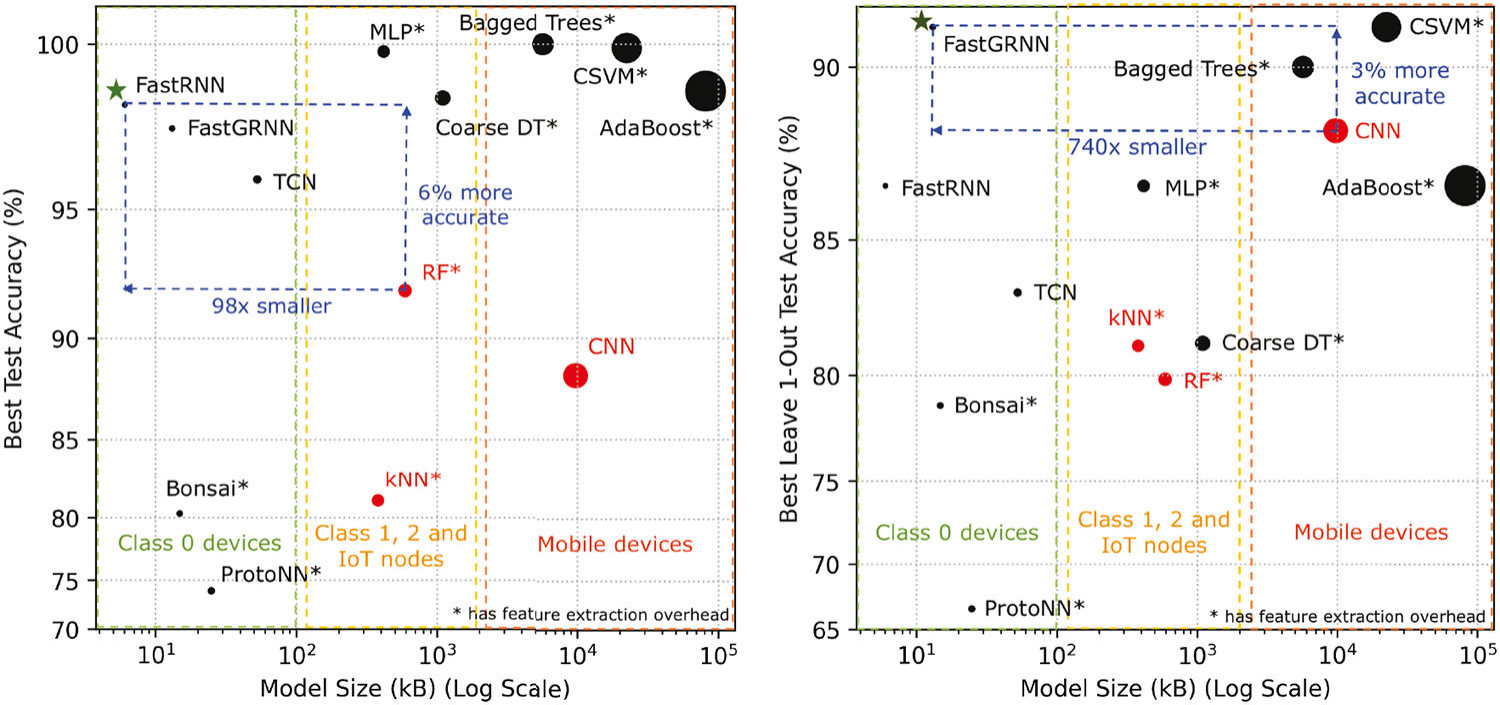
Performance comparison of our earable activity detection models (colored black) versus proposed models in literature (colored red). For activity detection on seen participants, FastRNN provides 6% accuracy improvement over the SOTA, while being 98× smaller. For activity detection on unseen participants, FastGRNN provides 3% accuracy improvement over the SOTA, while being 740× smaller. Both classifiers are suitable for deployment on ultra-resource-constrained devices.

Fig. 6 showcases the accuracy and model size of our earable activity detection models (colored black) versus proposed models in literature (colored red), namely CNN [38], RF [75], and kNN [38]. For activity detection on seen participants, compared to the SOTA RF model, FastRNN is 98× smaller and 6% more accurate without needing additional feature extraction overhead. For activity detection on unseen participants, compared to the SOTA CNN model, FastGRNN is 740× smaller and 3% more accurate without needing additional feature extraction overhead. In addition, our lightweight models are suitable for implementation on devices on Class 0 devices (Internet-of-Things (IoT) devices with < 100 kB flash [47]), while the SOTA, as well as our conventional classifiers, can only be run on mobile devices or Class 1 (IoT devices with ~ 100 kB flash [47]) and Class 2 (IoT devices with ~ 250 kB flash [47]) devices. Using our model zoo, it is possible to generate activity detection models suitable for a broad spectrum of hardware classes with different compute constraints while maintaining superior accuracy.

### 5.2 Activity Detection Multiclass Metrics and Effect of Window Size

**Fig. 7.**
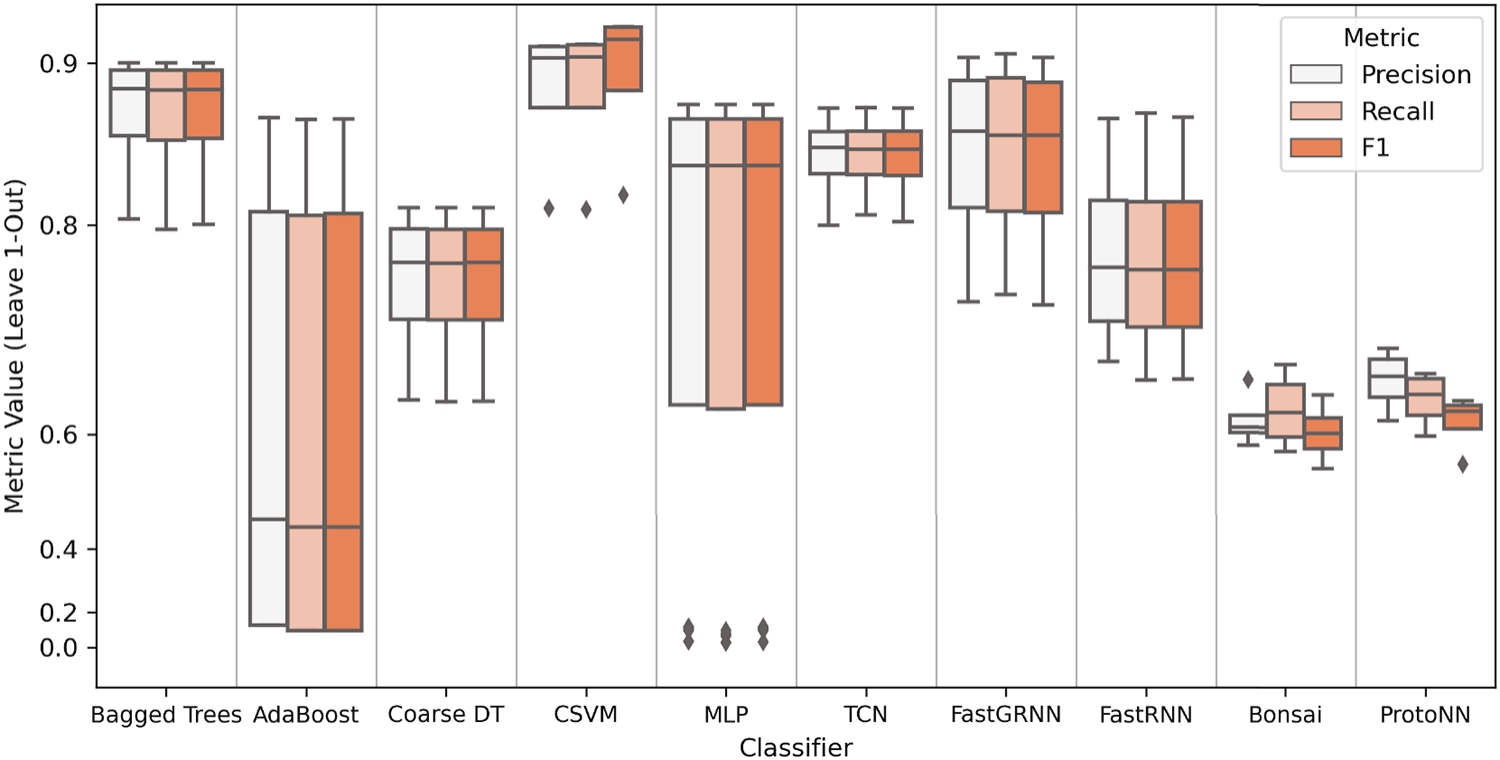
Boxplot showing leave-1 out multiclass error metrics for our proposed classifiers for varying window sizes.

Fig. 7 outlines the leave-1 out class-dependent errors (precision, recall, and F1 score) for all 10 activity classifiers. The multiclass metrics were obtained for different window sizes (1, 3, 5, and 10 seconds for conventional classifiers; 1, 2, 3 and 5 seconds for lightweight classifiers). From Fig. 7 (left), we can see that the median precision and recall of each classifier are roughly similar. We can also observe that SVM, TCN, Bonsai, and ProtoNN are the most stable in terms of multiclass classification quality across different window sizes. The SVM classifier achieves the highest median precision, recall, and F1 score, indicating a high degree of completeness and exactness and a low number of false positives and false negatives across classes. On the other hand, AdaBoost and MLP have the largest range of class-dependent error for different window sizes, indicating a significant dependence of accuracy on window size.

**Fig. 8.**
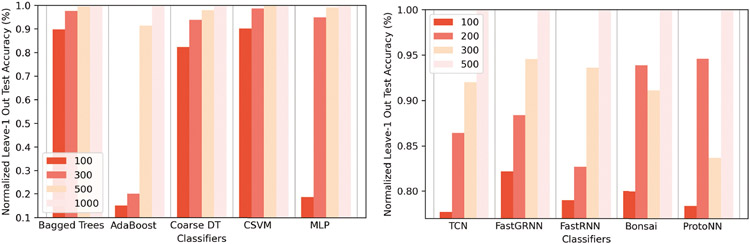
Effect of window size on normalized leave-1 out test accuracy on conventional and lightweight activity classifiers.

Fig. 8 shows the normalized leave-1 out test accuracies for all 10 classifiers with varying window sizes. The accuracy of all classifiers improves with larger window sizes. This is because, with larger time windows, the classifier has access to more spatial and temporal information. Small windows may not always have enough differential features to classify each activity separately. However, time windows longer than 2.5-3.5 seconds [97] may not be helpful when rapid changes in activities occur or when a macro-activity can be decomposed into transient micro-activities. Furthermore, longer time windows can reduce inference speed [97]. Thus, for practical deployment, it is recommended to keep window size around 2.5-3.5 seconds [97].

### 5.3 Activity Detection Cross-Validation Studies (Leave-n Out)

**Fig. 9.**
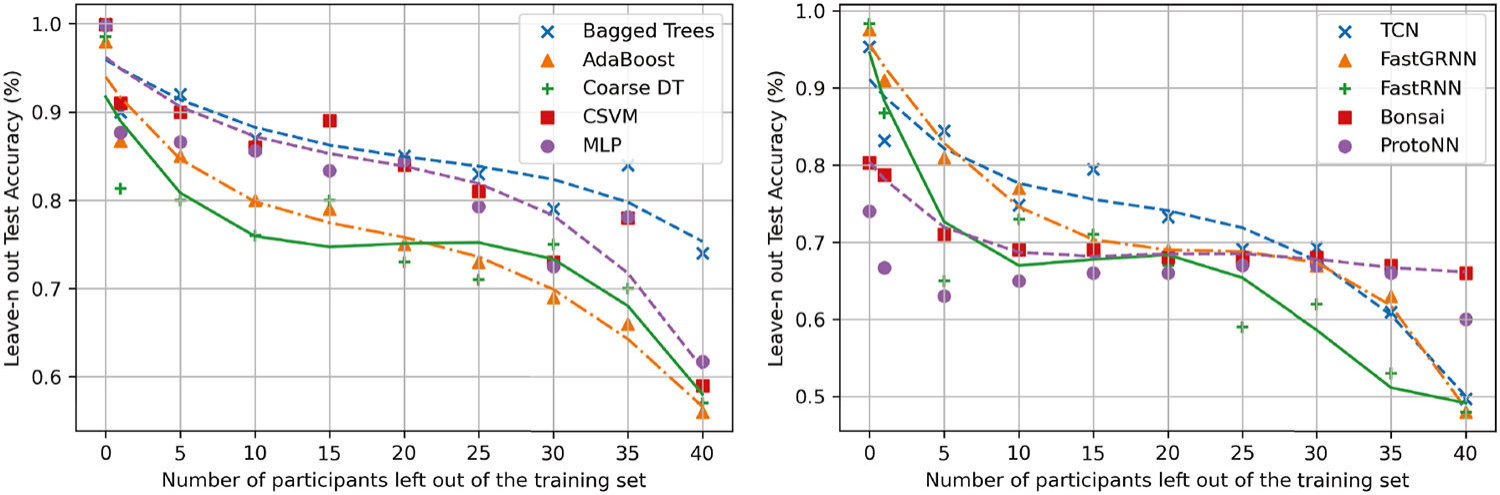
Generalization performance (leave-n out test accuracy) of our earable activity classifiers with increase in the number of participants left out of the training set. The window size was 10 seconds for conventional classifiers and 5 seconds for lightweight classifiers.

To test the generalization capability of all 10 classifiers with a varying number of unseen participants in the training set, we performed a leave-n out study where we tested the accuracy of all the classifiers with a varying number of participants left out of the training set. Fig. 9 showcases summarizes the results of the study. While the test accuracy on unseen participants drops with an increase in the number of participants left out of the training set, the accuracy of lightweight classifiers drops by around 11.8% more on average over conventional classifiers for the same value of n. As discussed in Section 5.1, lightweight models suffer from generalizability due to a low number of redundant weights to model global features. The problem is particularly worse for FastRNN, FastGRNN, and TCN, which attempt to make inferences on raw data, compared to Bonsai and ProtoNN, which make inferences on features. FastRNN, FastGRNN, and TCN require the injection of domain adaption, possibly via domain adversarial training to make these NN robust across domains [16, 30] if they are to work without feature extraction.

### 5.4 Performance of Hardware-in-the-loop Bayesian Neural Architecture Search

**Fig. 10.**
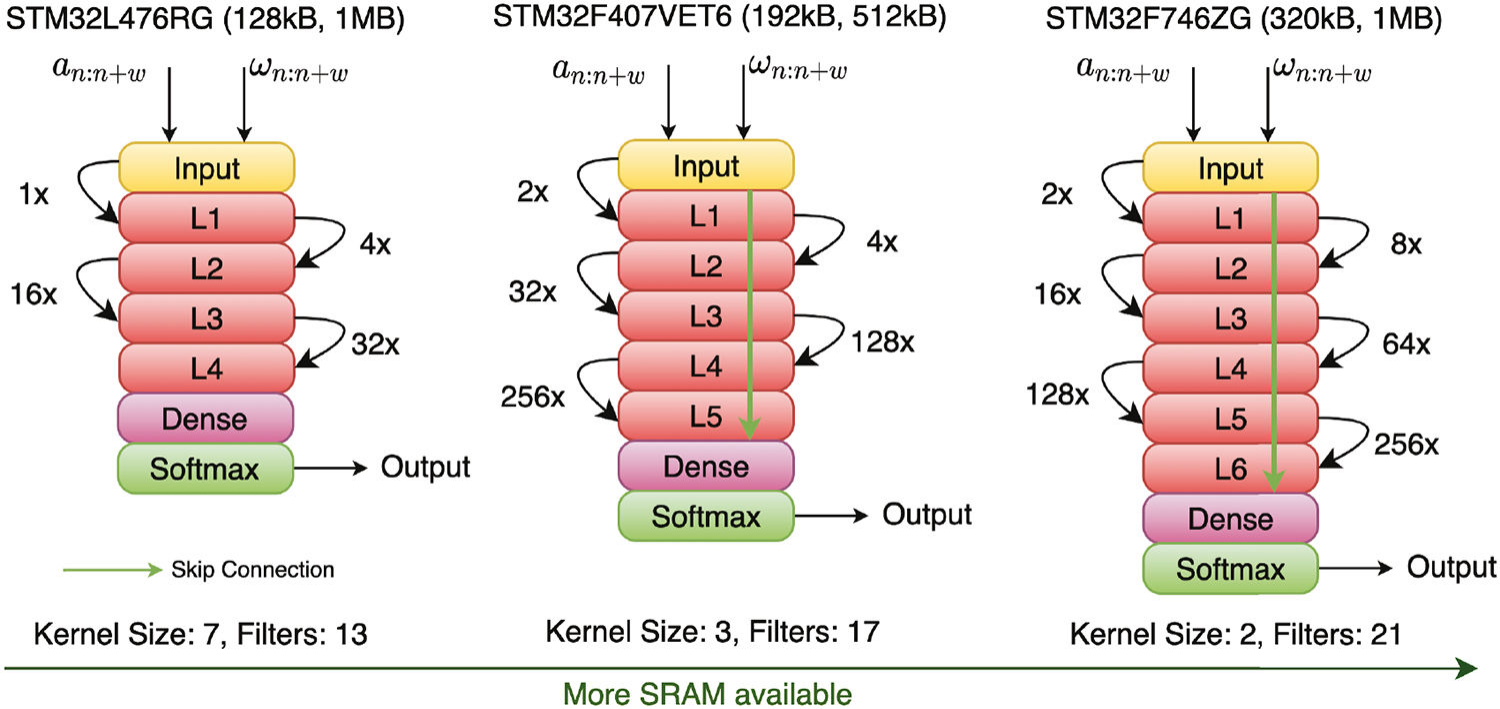
Example architectural adaption and device capability exploitation by Bayesian NAS based on resource usage for TCN activity classifier. The RAM and flash constraints of the device are written inside parenthesis. *L_*i*_* refers to *i*^th^ layer of TCN.

To showcase how our NAS helps adapt the same model for different hardware, we optimized the TCN model for five different hardware with different compute capabilities. Fig. 10 illustrates how our hardware-aware NAS tunes the TCN architecture for three of those hardware to improve model accuracy by maximizing the available compute resources of the device. As the SRAM capacity of the device increases, the NAS framework increases the number of layers and filters in the TCN model. To prevent EVGP, NAS also adds skip connections as the number of layers increases. Another interesting observation is how our NAS pipeline assigns the dilation factor to each layer. To capture both local and global dynamics within a limited computing budget, NAS assigns a small dilation factor to the lower layer to capture short-term local context, and a large dilation factor in higher layers to capture long-term global inter-dependencies in the temporal sequence. Classically, a human designer would assign dilation factors that increase by a constant factor with each successive layer instead of the complex dilation factor assignment that the NAS performs. This observation further strengthens the need for intelligent AutoML frameworks for deployable ML model development.

**Fig. 11.**
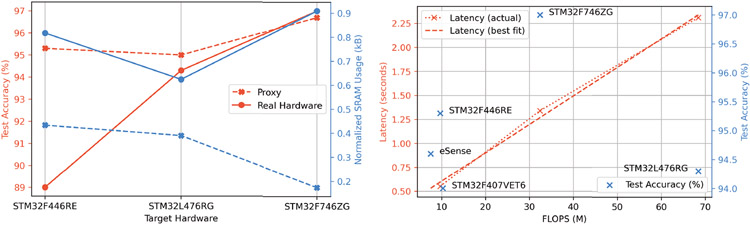
(Left) Accuracy and SRAM usage estimation comparison between proxyless Bayesian NAS and proxied Bayesian NAS for different devices. The SRAM usage is normalized by maximum RAM capacity of each device. (Right) Relationship between FLOPS, model latency and accuracy for TCN earable activity classifiers geared towards different devices. FLOPS and latency have a strong linear correlation.

We also performed an ablation study to see how proxyless (with real-hardware) and proxied versions (with proxy to simulate hardware metric) of our NAS framework differ in performance with three real hardware devices. The results are shown in Fig. 11 (left). From Fig. 11 (left), we can observe that as the resource budget of target hardware loosens, the difference in accuracy between the best performing model found by proxyless NAS and proxied NAS reduces. In both cases, the accuracy of the model improves with more capable hardware. The difference in accuracy, albeit within ±6%, arises from the runtime interpreter and RTOS overhead which the proxy for SRAM and flash fails to account for. As a result, some well-performing model candidates found by proxied NAS may not fit on the real hardware when one takes We can infer this from the offset observed between SRAM usage reported by proxyless and proxied NAS. Overall, HIL becomes important for ultra resource-constrained devices, where all overheads need to be accounted for.

Besides quantifying the difference between proxyless and proxied NAS for memory and accuracy modeling, we also studied the relationship between FLOPS, model latency (from real hardware), and model accuracy, summarized in Fig. 11 (right). We observed that there is a strong positive correlation (Pearson Coefficient, *ρ* = 0.998) between FLOPS and model latency, indicating that it is possible to develop an analytical model correlating FLOPS and model latency without requiring HIL. The same observation was made by Banbury *et al*. [8] for models geared towards microcontrollers. Analytical models for latency will be much faster over getting the latency metric directly from real hardware [78]. However, we found that more FLOPS do not always translate to higher model accuracy. We did not observe a significant correlation (*ρ* = 0.0107) between FLOPS and model accuracy, which was also observed in Table 9. As discussed earlier, only a small portion of model parameters are responsible for contributing towards model accuracy, resulting in no correlation between FLOPS and classification performance.

### 5.5 Head-Pose Filter Size and Error Characteristics

**Fig. 12.**
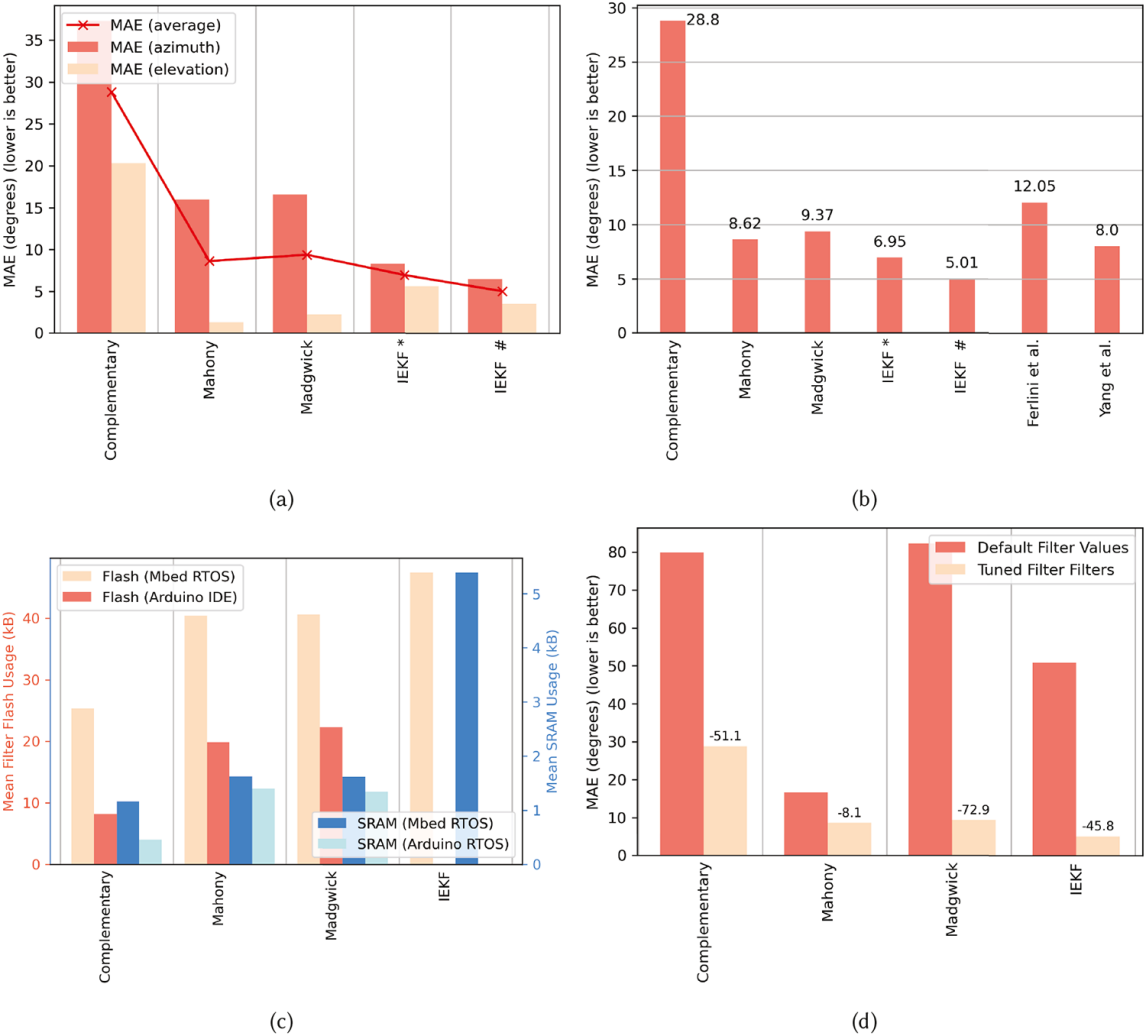
(a) Error characteristics (planar and average) of the head-pose filters (tuned) in filter zoo . IEKF* refers to IEKF with translational movements and IEKF# refers to IEKF with only head movements. (b) Comparison of error characteristics of proposed head-pose filters against SOTA (c) SRAM and flash usage of proposed filters for ARM Cortex M4 (running Mbed) and AVR RISC (running Arduino) processor architectures (d) Error reduction via BO of filter parameters.

Fig. 12 summarizes the error characteristics and resource usage of the head-pose filters in filter zoo. From Fig. 12 (a), we can observe that the IEKF provided the lowest mean absolute error (MAE) of 6.49° and 3.53° for head-tracking in the azimuth and elevation plane, respectively. This is expected, as the IEKF can minimize the variance in the attitude estimate optimally through the innovation and estimate covariance matrices. Compared to Yang *et al*. [102], the IEKF provides 1.6× improvement in error characteristics as shown in Fig. 12 (b) using a single earable IMU. Note that the average MAE of the IEKF increases by ~ 2° when translational motion artifacts (e.g., walking) are introduced along with head movements. Although the IEKF provides superior error characteristics over other filters, it is also the most resource-intensive as shown in Fig. 12 (c). Compared to Madgwick or Mahony filter, the IEKF requires 17% more flash and 3.3× more SRAM when implemented for Mbed RTOS, while providing 1.8× lower MAE. Given the resource usage, IEKF cannot be implemented on AVR RISC processors running Arduino. A trade-off would be to choose the Mahony filter, which requires an average of 1.5 kB of SRAM while providing an MAE of 8.62°. The filter requires 19.8 kB and 40.4 kB of flash when implemented on Cortex-M4 and AVR RISC microcontrollers, respectively. The lightest of all filters is the complementary filter, which, unfortunately, also suffers from the largest MAE, as it cannot account for drift and noise as elegantly as other filters. Note that since these filters are analytical, they are wearer-independent, unlike the activity detection models.

Fig. 12 (d) showcases the importance of tuning filter parameters. Without optimization, the average MAE of the filters increases anywhere between 8.1° and 72.9°. As a result, before using the filters in the filter zoo for head-pose estimation, it is recommended to calibrate the filters on some samples of the wearer’s head movements. We suggest the following orientation filter calibration program:

- First, the accelerometer and the gyroscope within the earable must be calibrated to remove static gyroscope bias drift and estimate the accelerometer gains and biases using the techniques described in Section 3.2.
- The user immediately wears the earable after calibration, then faces roughly straight ahead (azimuth angle of 0°) and starts IMU data logging, all while not making any significant head movements. The wearer then moves the head slowly from 0° to any angle larger than 10° but less than 90° to the right, and back. Note that the user does not have to move the head exactly back to the initial azimuth angle of 0°.
- The wearer repeats the process but for the elevation plane (vertical head movement). Note that we suggest the user complete the two data logging steps within a minute of performing accelerometer and gyroscope calibration.
- Since the gyroscope and the accelerometer has just been calibrated, we can obtain the 3D ground truth orientation (roll (*ϕ*_*t*_), pitch (*θ*_*t*_), and yaw (*ψ*_*t*_)) for the user’s head trajectory at timestep *t* directly from the IMU readings using the following equations:
(15) $${\left[\begin{array}{c} \phi_{t}\\ \theta_{t} \end{array}\right]}_{a}=\left[\begin{array}{c} \text{arctan}\left(\frac{A_{y,t}}{\sqrt{A_{x,t}^{2}+A_{z,t}^{2}}}\cdot \frac{180}{\pi }\right)\\ \text{arctan}\left(\frac{A_{x,t}}{\sqrt{A_{y,t}^{2}+A_{z,t}^{2}}}\cdot \frac{180}{\pi }\right) \end{array}\right],\;{\left[\begin{array}{c} \phi_{t}\\ \theta_{t}\\ \psi_{t} \end{array}\right]}_{\omega }=\left[\begin{array}{c} \phi_{t-1}\\ \theta_{t-1}\\ \psi_{t-1} \end{array}\right]+\left[\begin{array}{c} \omega_{x,t}\\ \omega_{y,t}\\ \omega_{z,t} \end{array}\right]\left[\begin{array}{c} f_{s}^{-1}\\ f_{s}^{-1}\\ f_{s}^{-1} \end{array}\right]$$
(16) $${\left[\begin{array}{c} \phi_{t}\\ \theta_{t}\\ \psi_{t} \end{array}\right]}_{\text{GT}}=\left[\begin{array}{c} 0.5(\phi_{t,a}+\phi_{t,\omega })\\ 0.5(\theta_{t,a}+\phi_{t,\omega })\\ \psi_{t,\omega } \end{array}\right]$$
  where, *A*_*x*_,_*y*_,_*z*_ refers to accelerometer readings, *ω*_*x*,*y*,*z*_ refers to gyroscope readings, and *f*_*s*_ refers to IMU sampling rate.
- The filter parameters can then be tuned by plugging in the recorded IMU data and the ground truth orientation and performing an exhaustive search over the possible range of filter parameters to minimize filter MAE.

The orientation program does not cause any hassle to the user as the user does not have to follow any strictly bounded head motion trajectory. In theory, the number of data points required for calibration equals the number of tunable and initial filter parameters (e.g., Madgwick and complementary filters have only a single tunable parameter, Mahony filter has two tunable parameters, while the IEKF has 4 tunable parameters and a 9×9 initial process noise covariance matrix). More data points can help provide a global and over-parametrized notion to the exhaustive search, possibly yielding better optimal values of the filter parameters. However, the search will be slow if the number of points is too large. Furthermore, if the user attempts to collect the orientation filter calibration data over a long time period, then the initial static IMU calibration parameters will become invalid. Therefore, more data points will not essentially lead to better estimates of filter parameters.

## 6 APPLICATIONS AND CASE STUDIES

To highlight the utility of Auritus, we showcase three canonical applications developed by using the tools provided in Auritus. These include fall detection (Section 6.1), spatial audio generation (Section 6.2) and interacting with objects in an AR digital twin (Section 6.3).

### 6.1 Fall Detection

**Fig. 13.**
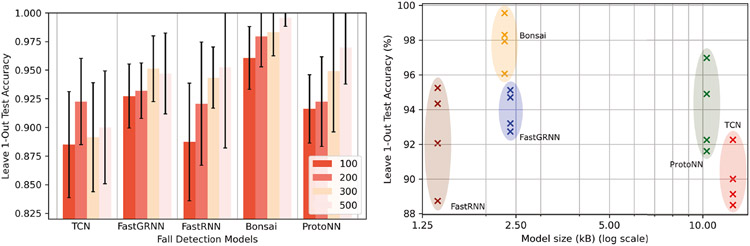
(Left) Leave-1 out fall detection accuracy of lightweight ML models for various window sizes trained to distinguish between falls and non-falls. (Right) Model size versus leave-1 out fall detection accuracy of lightweight ML models trained to distinguish between falls and non-falls.

Falls cause frequent injuries and death among the elderly population, with ~684,000 fatal cases occurring annually [1, 42, 101]. With an increasing number of elderly people living alone [90], there is a strong association between living alone and suffering from a fall (*χ*^2^ = 0.005) [23] among senior citizens, with 37 million cases requiring medical attention [1]. Thus, it is necessary to develop an accurate yet lightweight and real-time fall detection system that can reduce the lead time between detection of a fall and receiving medical attention [90, 101]. Thereby, we designed ultra-lightweight ML models using Auritus that can distinguish between falls and non-falls through earables. Fig. 13 illustrates the performance of fall detection in terms of model size and leave-1 out test accuracy for various window sizes. In general, the leave-1 out accuracy of fall detection models improved with larger window sizes. Among all the models, Bonsai and ProtoNN had the highest average leave-1 out fall detection accuracy of 99% and 98%, respectively for 5-second windows. We managed to squeeze the model size to only 2.3 kB for Bonsai using BO. The model size is so small that Bonsai requires negligible resources to be run in real-time on microcontroller-class devices. Bonsai can also maintain its accuracy within ±1% for window sizes smaller than 5 seconds (e.g., 2 and 3 seconds). Small window sizes are important for fall detection as the essential part of the fall event typically lasts around 2 seconds [58].

### 6.2 Binaural Audio Rendering

Spatial audio refers to the process of generating audio that provides the listener with a perception about the direction, distance, size, and type of object [25]. Also known as binaural audio, 3D audio is useful for indoor acoustic AR [102], providing directional localization cues [19], exercise feedback [77], and interacting with virtual objects [71]. Given the small size and portability of earables coupled with the presence of both head-tracking sensors and stereo speakers, it is possible to generate a lightweight perception-processing-feedback setup by combining head-tracking with binaural audio. Generally, a head-pose filter supplies azimuth and elevation angles to a pair of two head-related transfer functions (HRTF) [17, 102]. The HRTF is the response of how the human ear perceives the location of the sound. Mono audio is convolved with the HRTF finite impulse response filters to generate binaural audio, with the interpolated HRTF calculated at the position of the head-tracked.

**Fig. 14.**
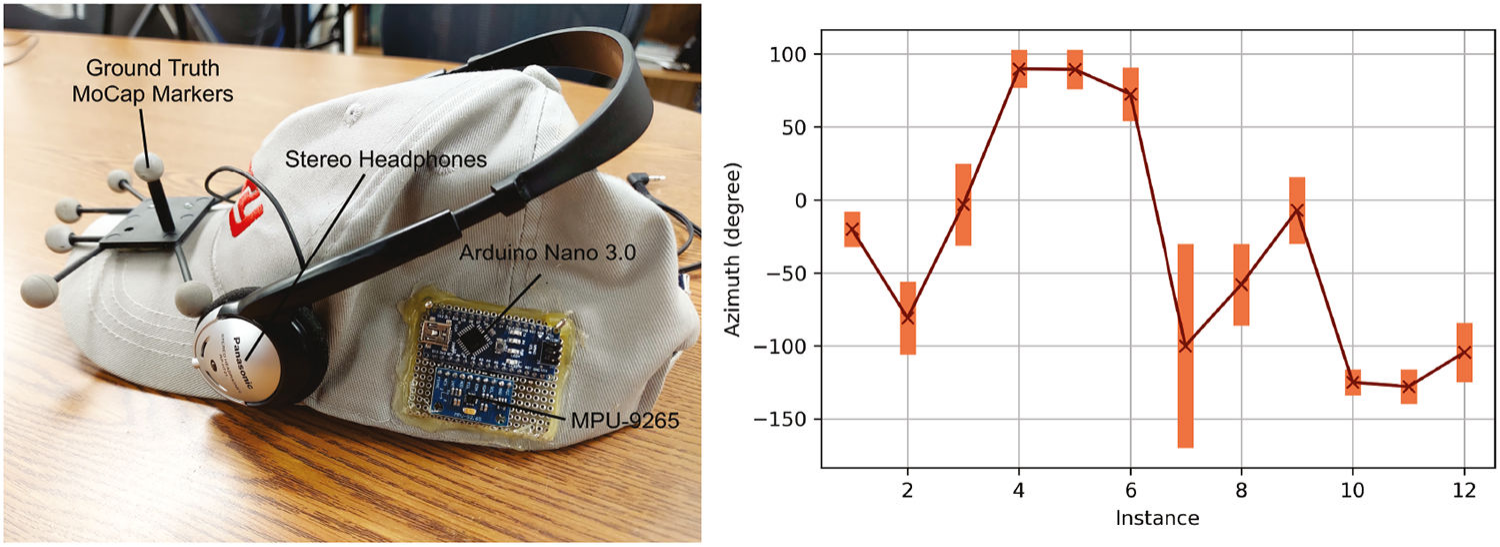
(Left) Hardware setup for binaural audio rendering experiment. (Right) Snapshot of sound source localization test with real participants to quantify head-pose filter performance.

We implemented a 3D spatial audio framework in MATLAB using Auritus to showcase the utility of our head-tracking filters. We used the ARI HRTF database [17]. The database has data points for 1550 positions for over 200 subjects, with an azimuthal resolution of 2.5° (−45° to +45°) and elevational resolution of 5° (−30° to +80°) [17]. The HRTF for a point outside the angular range in the dataset is found using interpolation. For head tracking, we used the IEKF. Since eSense is non-programmable, we created our own hardware setup to stream head-tracking data in real-time to the HRTF, shown in Fig 14 (left). The setup consists of an Arduino Nano 3.0 connected to an MPU-9265 9DoF IMU. We only used the accelerometer and gyroscope data from the 9DoF IMU as the original earable does not have a magnetometer. We also calibrated the accelerometer and gyroscope of the MPU-9265 using calibration techniques described in Section 3.2. The audio is streamed through headphones from the HRTF kernel.

Fig 14 (right) shows a snapshot for the binaural audio sound source localization test. In the test, we asked a participant to roughly locate which azimuth direction a virtual sound source is situated at by listening to spatial audio using our hardware setup. It is generally assumed that the human sound source localization resolution using binaural cues varies widely around ±6 − ±20° [22, 70], and a head-pose filter must be able to provide the direction of the head with an MAE less than 20°. From our sound source localization test, we obtained a localization error of ±22.7°. The error is on the higher end of the localization spectrum not due to IEKF errors, but partly due to the use of non-personalized HRTF database [102]. In addition, some of the binaural cues were outside the −45° to +45° azimuth range provided by the dataset, causing errors to be introduced in spatial sound generation due to interpolation of HRTF kernel. The participant we selected for the test may have had an aural localization resolution around ±20°, which added to the cumulative errors from the HRTF database.

### 6.3 Interacting with AR Frameworks

**Fig. 15.**
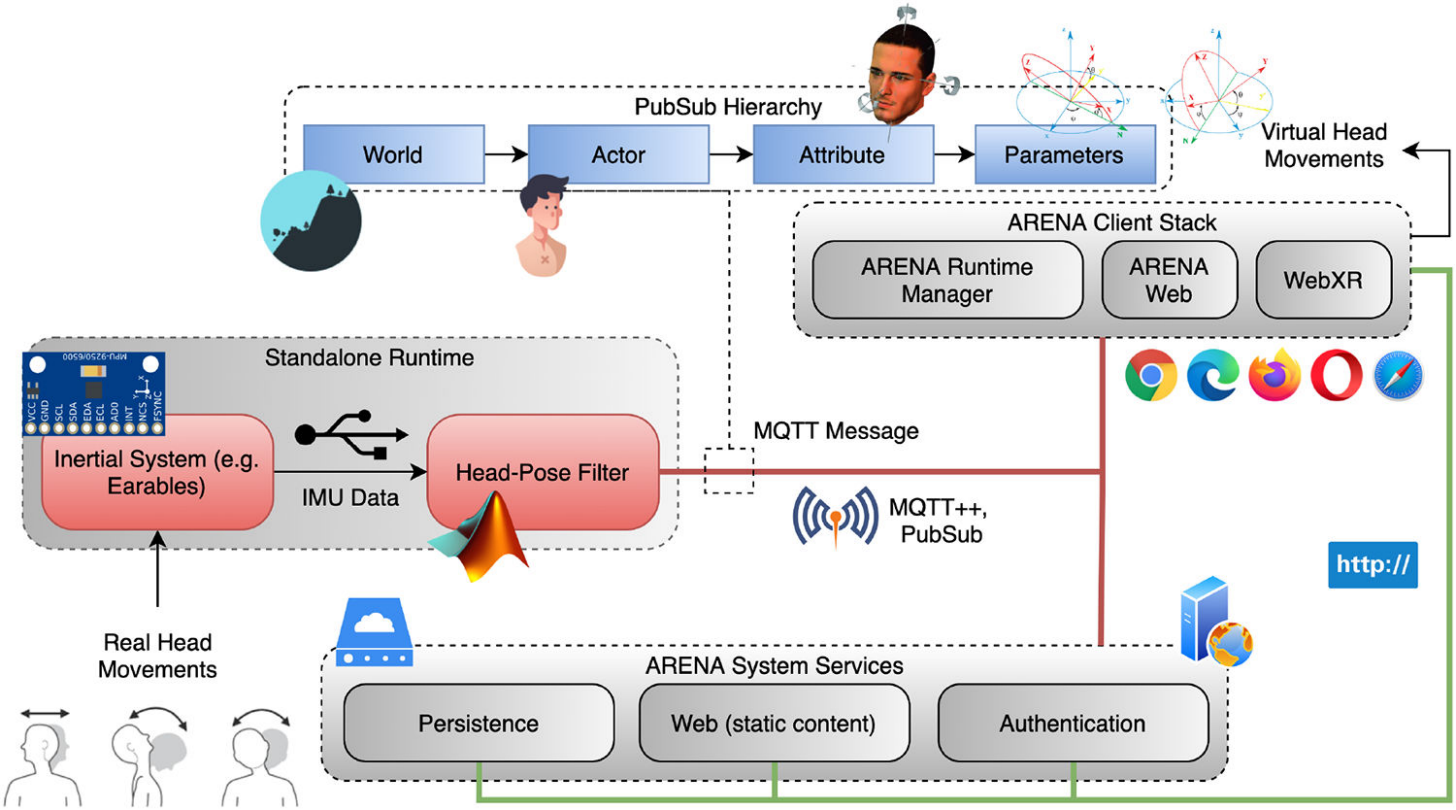
Controlling head-orientation of virtual actors in an AR space (CONIX ARENA) using real head-pose. The head-pose filter application communicates with the AR framework through Pub/Sub topic hierarchy, which in turn alters atrribute parameters (in this case, head pose) of target actor in the AR world.

One of the most promising applications of earables is the ability to control virtual objects an AR digital twin [19]. We used the same hardware setup developed in Section 6.2 to control the orientation and motion of actors in a virtual world wirelessly using head movements. Fig. 15 showcases the interaction between the AR framework and the head-pose application. We used the open-source CONIX Arena [71] AR architecture to showcase this application. The head-pose application communicates with the virtual world using MQTT (Pub/Sub) messages. The Pub/Sub message specifies the ARENA server to connect to, the realm (world), an object ID within the realm specifying the actor, the attribute (e.g., head orientation) to alter, and the values of the parameters of that attribute (e.g., Euler angles). We observed negligible delays in updating the parameters through MQTT, with the head-pose filter (an IEKF) running at 100 Hz. The framework thus allows one to control the parameters of virtual actors using real sensor values in the real world in near real-time without significant latency. Although we developed the application to control the head-pose of virtual humans, it is also possible, for example, to control real drones, cars, and appliances using head-movements through their digital twin in the ARENA realm.

## 7 CONCLUSION, LIMITATIONS, AND FUTURE WORK

Given the commercialization potential of earables, an open-source end-to-end toolkit can enable the accelerated development of future ventures, catalyzing the adoption of new technologies and sensing modalities in smart earbuds [19]. Auritus provides a tightly-coupled collection of open-source and extendable libraries, datasets, and tools that allow application developers and researchers in earable computing to collect human movement data, label time-series data interactively, and develop new human movement models and filters. The model and filters in Auritus are designed to meet hardware constraints without sacrificing accuracy. In the process, Auritus advances the SOTA activity classification models and head-pose filters in their accuracy even with lightweight models and filters. Moreover, our experience with Auritus demonstrates that the toolkit is capable of supporting a variety of different applications and research needs. Several lessons, limitations and directions of future work for our framework are as follows:

- Since the activities in our dataset are scripted and of short periods, the evaluation has some limitations. *Firstly*, the activities are not completely natural and continuous due to missing context and context change. *Secondly*, due to short activity periods, the effects of earable placement and displacement are not significant. Both of these effects can provide erroneous classification results in the wild and lead to an upper bound in accuracy our system can reach.
- While our work shows that Auritus is capable of supporting diverse applications, our framework is currently limited to work with inertial sensor data only. Smart earbuds also include other modalities (e.g., audio, BLE, temperature, etc.), which can be disruptive for a number of physiological applications [19]. The largest change for such an expansion would be in the data collection and labeling pipeline. The smartphone application needs to be expanded to collect data for other modalities, while modality-specific ground truth data collection hardware (e.g., microphones, binaural audio generators, and bio-electrical and physiological signal measurement devices) need to be invested in. While the lightweight model generation pipeline generalizes to any modality without changes, any optional feature extraction and windowing need to be domain-specific (e.g., log-Mel spectrogram for audio, received signal strength indicator for BLE, and fast Fourier transform coefficients for heart rate).
- Our findings indicate that over-the-air model adaption is important to handle cross-user variations and domain variance for lightweight models, which our framework currently does not handle. This would require the earable devices to be capable of collecting human movement data and adapting the decision boundaries of the baseline model onboard on the fly to reduce performance drop [13, 55]. However, since earables are constrained in SRAM, more work needs to be done to allow efficient on-device training. Domain adversarial training may also be able to generate models robust to ambient disturbances and cross-user variations [16, 30].
- Although most commercial earables (including eSense) do not yet support firmware changes and only allow access to data, we speculate that future earables will allow onboard programming with apps specifically developed for onboard inference. For example, early wrist-worn devices were fixed worn devices with applications running on smartphones. Modern smartwatches now allow programming and on-device processing [18]. When such devices emerge in earable computing, Auritus would provide developers with the necessary model training and development framework.
- We found out that sensor data from earable devices suffer from missing data, cross-channel timestamp misalignment, and window jitter, due to packet drops and the absence of on-chip clocks. This can reduce the performance of ML models when training for complex event processing [84]. The solution can be to either inject ML models with uncertainty awareness via uncertainty-injected training pipeline [84, 89] or use onboard clocks and hardware enhancements for precise time-synchronization [88] and handling packet drops.

## Figures and Tables

**Fig. 5.**
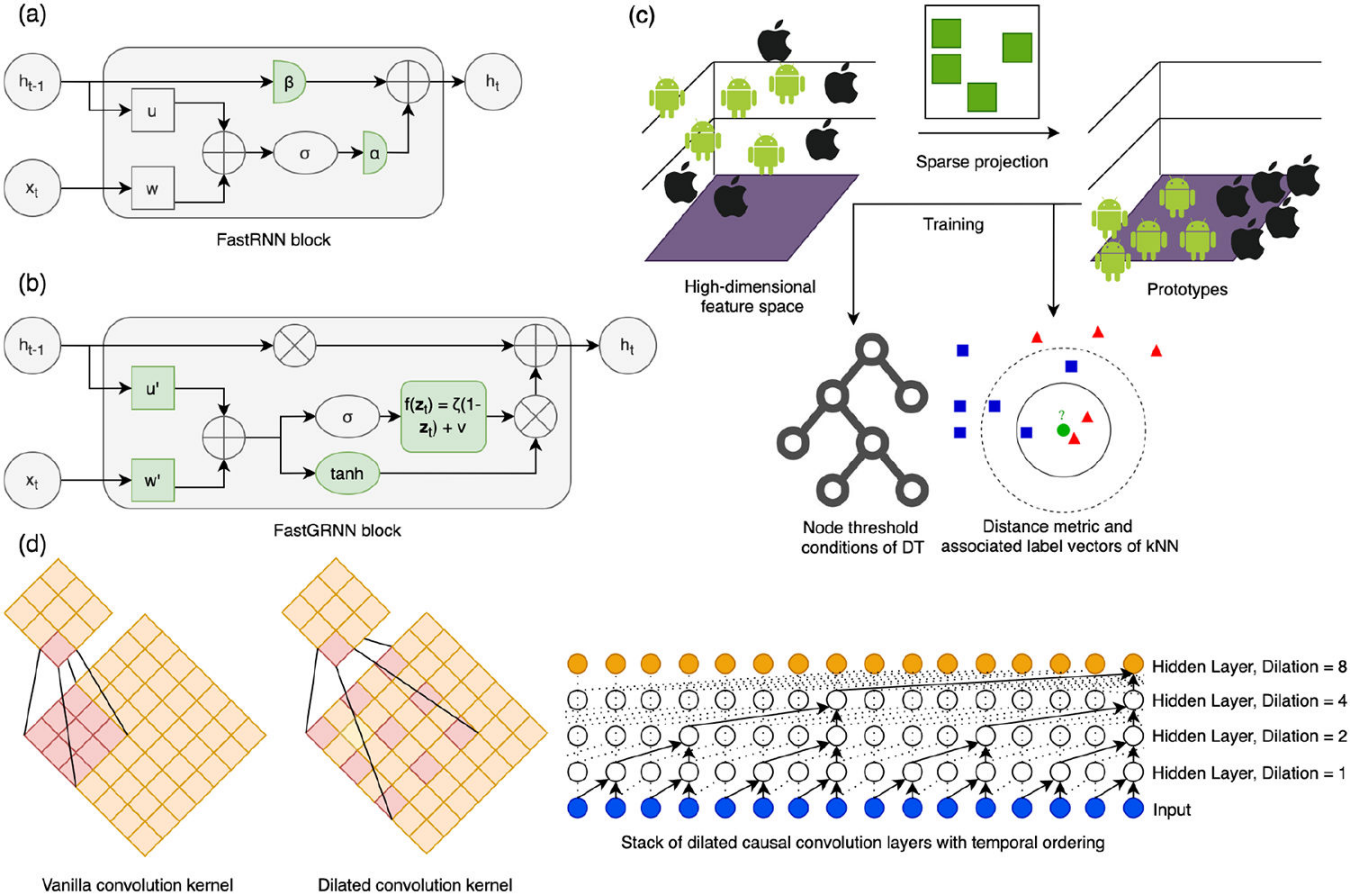
Illustration of lightweight model architectures geared towards TinyML devices. (a) The addition of a residual connection with two scalars (*α*, *β*) stabilizes vanilla RNN training while taking advantage of the relative lightweightness of vanilla RNN against gated RNN. (b) Converting the residual connection to a gate while enforcing **U** and **W** to be LSQ yields lightweight yet accurate gated RNN. (c) Sparsely projecting input features to a low-dimensional space allows DT and kNN to be computationally efficient. (d) Enforcing causal convolution and dilated kernels allows spatial and temporal feature extraction in long time-series sequences without requiring recurrent connections or significant compute.

**Table 1.** Performance metrics of existing activity detectors and head-pose filters for earables

Activity Detectors (Full Body)			
*Classifier*	*Accuracy* (%)	*Feature Extraction*	*Model Size* (kB)
CNN [38]	88.3	No	9758
kNN [38]	81.2	Yes	381
Random Forest (RF) [75]	92.94	Yes	593
K-means Clustering [66]	96.8	Yes	-

Head-Pose Filters			
*Filters*		*Error characteristics*	
Ferlini *et al*. [28]		5.4 degrees (short) and 18.7 degrees (long)	
Yang *et al*. [102]		~8 degrees (after 3 rounds of rotations)	

**Table 2.** Summary of executed activities.

	ADL	Description
1	Walking (W)	The participant is asked to walk forward in a straight line, make a turn (clockwise or anti-clockwise) at the end of a corridor and repeat (average speed: 1.5-3.5 mph).
2	Jogging (R)	Similar to walking, but the participant runs slowly at uniform pace instead of normal walking pace (average speed: 3.0-5.0 mph).
3	Jumping (J)	Each participant jumps at a particular spot without translational motion.
4	Standing (St)	The participant stands freely with true-to-life head and limb movements allowed.
5	Turning Left (Tl)	The participant walks forward in a counter-clockwise circle.
6	Turning Right (Tr)	The participant walks forward in a clockwise circle.
7	Sitting (Si)	Each participant is asked to sit on an office chair freely, allowing for natural limb and head movements.
8	Lying (L)	Each participants lay on a sofa in various common sleeping positions.
9	Falling (F)	The participant falls freely on a padded sofa from a standing position.

**Table 3.** Results of Kolmogorov-Smirnov test on dataset participant statistics.

Parameter	DF	Statistic	P > D	Inference
Age (yrs)	45	0.1271	0.37217	Can’t reject normality at 0.05 significance level
Height (m)	45	0.13309	0.30585	Can’t reject normality at 0.05 significance level
Weight (kg)	45	0.14081	0.43016	Can’t reject normality at 0.05 significance level
Ear height from origin (inches)	45	0.11569	0.55641	Can’t reject normality at 0.05 significance level

**Table 4.** Results of Kruskal-Wallis ANOVA between DTW distances of human motion from same class and different classes.

Motion Type	*χ* ^2^	P > *χ*^2^	Inference
Activity	6.96337	0.00832	At the 0.05 level, the populations are significantly different
Head-Pose	5.56564	0.01832	At the 0.05 level, the populations are significantly different

**Table 5.** Features extracted for Bonsai, ProtoNN, and the five conventional activity classifiers. Only the shaded features were used for Bonsai and ProtoNN.

Dominant Sign	Entropy	Integration	Interquartile Range
Kurtosis	Mean Abs. Dev.	Maximum	Minimum
Mean	Avg. Mov. Mean	Avg. Mov. Med.	Avg. Mov. Max.
Avg. Mov. Min.	Avg. Mov. SD	Avg. Mov. Var.	Avg. Mov. MAD
Autocorrelation	Avg. Vec. Norm	Avg. Z Score	Median
Norm	Pearson CC	Range	Skewness
Slope Sign Change	Signal Mag. Area	Standard Deviation	Variance
Variation	Zero Crossing	Time Window	

**Table 6.** List of hardware evaluated for NAS.

Hardware	SRAM (kB)	Flash (kB)	Proxy/HIL
Qualcomm CSR8670 (eSense platform)	128	16000	Proxy
STM32F446RE	128	512	HIL, Proxy
STM32F407VET6	192	512	Proxy
STM32L476RG	128	1024	HIL, Proxy
STM32F746ZG	320	1024	HIL, Proxy

**Table 7.** Design space for all 10 models in the model and filter zoo. The classifiers marked (F) require feature extraction overhead.

Candidate Model	Design Space (Ω)	HIL	Other Parameters (Fixed)
Bagged Trees (F)	Number of learners: 10-500	✗	✗
	Maximum number of splits: 1-23401		
	Number of predictors to sample: 1-241		
AdaBoost (F)	Number of learners: 10-500	✗	✗
	Maximum number of splits: 1-23401		
	Learning rate: 0.001-1		
Coarse DT (F)	Maximum number of splits: 1-23401	✗	✗
	Split criterion: {Gini, Twoing, Deviance Reduction}		
SVM (F)	Kernel: {Quadratic, Cubic, Linear}	✗	✗
	Multiclass method: One-vs-All, One-vs-One		
	Box constraint level: 0.001-1000		
	Standardize data: true, false		
MLP (F)	Number of hidden units: {15, 20, 50, 100}	✗	Number of layers: 2

TCN	Number of filters: 2-64	✓	Number of stacks: 1
	Kernel size: 2-16		Dropout: 0.0
	Use residual: true, false		Activation: ReLU
	Number of layers: 3-8		Normalization (weight, batch, layer): False
	Dilation factors: [1,2,4,8,16,32,64,128,256]		Learning Rate: 0.001 (Adam)
Fast GRNN	Hidden Units: 20-60	✗	Learning Rate: 0.01
			Decay Step and Rate: 200, 0.1
			Sparsity (U, W): (1.0, 1.0)
Fast RNN			
			Nonlinearity (update, gate): (tanh, sigmoid)
			Rank (U, W): (Full, Full)
Bonsai (F)	Sigmoid Parameter: 1-4	✗	Regularization (Z, W, V, T): (0.0001, 0.001, 0.001, 0.001)
	Depth: 1-6		Sparsity (Z, W, V, T): (0.2,0.3,0.3,0.62)
	Projection dimension: 10-70		Learning rate: 0.01
ProtoNN (F)	Projection dimension: 10-70	✗	Regularization (W, B, Z): ( 0.000005, 0, 0.00005)
	Number of prototypes: 10-70		Sparsity (W, B, Z): (0.8,1.0,1.0)
	*γ*: 0.0015-0.05		Learning rate: 0.03

**Table 8.** Best performance of conventional ML activity classifiers on our dataset. The optimal window size was 10 seconds for all classifiers.

Classifier	Optimal Hyperparameters	Test Accuracy (%)	Leave 1-out Test Accuracy (%)	Model Size (kB)
DT ensemble	Learners: 237, Splits: 23019, Mode: Bagging	**100**	90.0±8.5	5700
	Learners: 344, Splits: 715, Learning Rate: 0.44 Mode: AdaBoost	98.7	86.7±9.5	81600
Coarse DT	Splits: 736, Criterion: Dev. Red., S.gate Dec. Splits: All	98.5	81.3± 11.4	1100
SVM (1-1)	Kernel: Cubic, Penalty Level: 26.5, Normalization: Yes	99.9	**91.0±5.4**	22500
MLP	Hidden Layer: 2, Hidden Unit: 50	99.8	87.7±8.2	**418**
	Hidden Layer: 2, Hidden Unit: 100	99.5	86.7±8.6	

**Table 9.** Best performance of lightweight ML activity classifiers on our dataset. The optimal window size was 5 seconds for all classifiers. Note that the results shown for TCN are using proxies.

Classifier	Optimal Hyperparameters	RAM (kB)	Flash (kB)	FLOPS (M)	Test Accuracy (%)	Leave 1-out Test Accuracy (%)	Energy (mW)
TCN	(*eSense*) Filters: 15, Kernel Size: 2, Dilations: [1, 2, 4, 8, 32, 128, 256], Skip Connections: No	39.3	52.8	7.52	94.6	80.0±9.4	-
	(*STM32F407VET6*) Filters: 17, Kernel Size: 3, Dilations: [2, 4, 32, 128, 256], Skip Connections: No	47.6	54.6	10.3	94.0	83.0±10.3	-
	(*STM32F446RE*) Filters: 18, Kernel Size: 2, Dilations: [2, 4, 8, 16, 32, 64, 128, 256], Skip Connections: Yes	55.4	73.4	12.3	95.3	83.2±9.7	116^
	(*STM32L476RG*) Filters: 13, Kernel Size: 7, Dilations: [1, 4, 16, 32], Skip Connections: No	49.9	53.3	10.1	95.0	82.0±14.4	50^
	(*STM32F746ZG*) Filters: 21, Kernel Size: 2, Dilations: [2, 8, 16, 64, 128, 256], Skip Connections: Yes	55.6	66.4	10.1	96.7	79.0±9.9	418^
Fast GRNN	Hidden Unit: 50	**~ 2**	13.12	-	97.6	**91.0±5.0**	**41**-133^#^
Fast RNN	Hidden Unit: 32	**~ 2**	**6.04**	-	**98.3**	86.7±3.10	**41**-133^#^

Bonsai	Depth: 3, Sigmoid Parameter: 1.0, Projection Dimension: 22	**~ 2**	14.8	0.0136	80.3	78.7±5.9	250^∨^
ProtoNN	Projection Dimension: 70, *γ*: 0.004, Prototypes: 70	**~ 2**	24.9	0.0174	74.0	66.7±8.4	275^∨^

^ EEMBC EnergyRunner™ benchmark [7], RTOS: Mbed, Interpreter: TFLM

# EEMBC EnergyRunner™ benchmark, RTOS: Arduino, Compiler: SeeDot [34], Hardware: STM32L476RG and STM32F446RE

∨ Monsoon Power Monitor, OS: Raspbian, Interpreter: TFL, Hardware: Broadcom BCM2711

## CCS Concepts:

**Human-centered computing** → **Ubiquitous and mobile computing systems and tools**; • **Computing methodologies** → *Machine learning*, • **Computer systems organization** → Embedded systems.

## Footnotes

1
https://github.com/nesl/auritus

2 IRB approved for public release. IRB number: 21-001253

3
https://www.esense.io/

4
https://optitrack.com/cameras/prime-17w/

5
https://optitrack.com/software/motive/

6
https://shop.leica-geosystems.com/buy/package/x3

7 Bonsai, ProtoNN, FastRNN, and FastGRNN do not have any validation step, so we transferred the validation data to the training set.

8
https://www.msoon.com/lvpm-software-download

9
https://github.com/mlcommons/tiny

10
https://www.st.com/en/evaluation-tools/stm32-nucleo-boards.html
